# Identification of Different Extracellular Vesicles in the Hydatid Fluid of *Echinococcus granulosus* and Immunomodulatory Effects of 110 K EVs on Sheep PBMCs

**DOI:** 10.3389/fimmu.2021.602717

**Published:** 2021-02-23

**Authors:** Jing Yang, Jin'en Wu, Yong Fu, Lujun Yan, Yating Li, Xiaola Guo, Yong'e Zhang, Xiaoqiang Wang, Yujuan Shen, William C. Cho, Yadong Zheng

**Affiliations:** ^1^State Key Laboratory of Veterinary Etiological Biology, Key Laboratory of Veterinary Parasitology of Gansu Province, Lanzhou Veterinary Research Institute, Chinese Academy of Agricultural Sciences, Lanzhou, China; ^2^Qinghai Academy of Animal Science and Veterinary Medicine, Qinghai University, Xining, China; ^3^School of Chemical and Biological Engineering, Lanzhou Jiaotong University, Lanzhou, China; ^4^National Institute of Parasitic Diseases, Chinese Center for Disease Control and Prevention, Shanghai, China; ^5^Key Laboratory of Parasite and Vector Biology, Ministry of Health, Shanghai, China; ^6^National Center for International Research on Tropical Diseases, Shanghai, China; ^7^World Health Organization Collaborating Center for Tropical Diseases, Shanghai, China; ^8^Department of Clinical Oncology, Queen Elizabeth Hospital, Hong Kong, China; ^9^Jiangsu Co-innovation Center for Prevention and Control of Important Animal Infectious Diseases and Zoonoses, Yangzhou, China

**Keywords:** *Echinococcus granulosus*, extracellular vesicles, sheep PMBC, hydatid fluid, 110 K EV

## Abstract

Echinococcosis, mainly caused by *Echinococcus granulosus*, is one of the 17 neglected tropical diseases. Extracellular vesicles (EVs) play an essential role in the host–parasite interplay. However, the EVs in the hydatid fluid (HF) of *E. granulosus* are not fully characterized. Herein, three different types of HF EVs, designated as 2 K, 10 K, and 110 K EVs based on the centrifugal force used, were morphologically identified. A total of 97, 80, and 581 proteins were identified in 2 K, 10 K, and 110 K EVs, respectively, 39 of which were commonly shared. Moreover, 11, 8, and 25 miRNAs were detected, respectively, and all of the 7 selected miRNAs were validated by qPCR to be significantly lower abundant than that in protoscoleces. It was further deemed that 110 K EVs were internalized by sheep peripheral blood mononuclear cells (PBMCs) in a time-dependent manner and thus induced interleukin (IL)-10, tumor necrosis factor-α (TNF-α), and IRF5 were significantly upregulated and IL-1β, IL-17, and CD14 were significantly downregulated (*p* < 0.05). These data demonstrate the physical discrepancy of three HF EVs and an immunomodulatory effect of 110 K EVs on sheep PMBCs, suggesting a role in immune responses during *E. granulosus* infection.

## Introduction

Echinococcosis, caused by the larval stage of the genus *Echinococcus*, is listed as one of the neglected tropical diseases by WHO (1, 2). Currently, there are at least eight recognized *Echinococcus* species, such as *Echinococcus granulosus sensu lato, Echinococcus multilocularis, Echinococcus oligathrus*, and *Echinococcus vogeli* (3, 4). Among them, *E. granulosus* and *E. multilocularis* are of major public health impact globally and are responsible for cystic echinococcosis (CE) and alveolar echinococcosis (AE), respectively (5–7). It was estimated that the global burdens by CE and AE were 285,500 disability-adjusted life years (DALYs) and 666,434 DALYs, respectively (2, 8). CE is a deleterious zoonosis, which has a great socioeconomic impact on public health and domestic livestock industry (9–12).

In the life cycle, *E. granulosus* requires two different hosts: an intermediate host (sheep) and a definitive host (dog) (13, 14). The adult resides in the small intestine of a definitive host, and the eggs are excreted with the host's feces into the surrounding environment. When a natural intermediate host incidentally ingests the food or water contaminated by the eggs, oncospheres hatch in the stomach and intestine and, then, pass through the portal and lymphatic vessels to reach the liver, lungs, and other organs through bloodstream circulation. They grow into unilocular cysts (metacestodes or hydatid cysts), which are filled with the hydatid fluid (HF) with a lot of protoscoleces. If a definitive host consumes infected organs or offal, the larva develops into the adult in the intestine and, thus, completes its life cycle (3, 9, 13).

In the HF, different active molecules, such as antigen B and antigen five that induced strong specific immune responses, have been found (10). Apart from these proteins, exosome-like extracellular vesicles (EVs) were also recently reported (7, 14). These EVs serve as a communicator to convey proteins, carbohydrates, lipids, mRNAs, and non-coding RNAs to recipient cells and are involved in numerous biological activities, such as cancer development, metastasis, immunity (15, 16), immunomodulation (17), bone remodeling (18), and others (19–25). EVs also play an essential role in host-parasite interactions by transferring virulence factors and effector molecules from parasites to hosts (26). There are several heterogeneous populations of EVs based on the size (27). Exosomes, a kind of smaller EVs, are phospholipid bilayer membranes, secreted from organelles with a size of 30~200 nm in diameter. Their biogenesis is initiated by inward budding of multivesicular endosomes, followed by the release *via* the fusion of multivesicular bodies with the plasma membrane (20, 26, 28). Several studies have demonstrated that helminth parasites secrete exosomes that play an essential role during infection (29). For example, *Heligmosomoides polygyrus*–derived exosomes inhibit innate immunity *in vivo* by suppressing the expression of the Type 2 cytokines, namely interleukin (IL)-5 and IL-13 (30). Similarly, *Toxoplasma gondii*–derived exosomes stimulate macrophage activation *via* the upregulation of pro-inflammatory cytokines (IL-12, IFN-γ, and TNF-α) and also produce partial protective immunity (31). Although exosome-like EVs were described in the *E. granulosus* HF (10, 14), not much is known about other HF EVs.

Herein, we firstly separated and identified three different EVs from *E. granulosus* (G1) HF. The protein and miRNA cargoes of all EVs were comparatively defined using high performance liquid chromatography tandem mass spectrometry (HPLC-MS/MS) and high-throughput sequencing, respectively. It was further demonstrated that 110 K EVs were internalized by sheep peripheral blood mononuclear cells (PBMCs) and, thus, induced the ectopic expression of cytokines and the core components in the LPS/TLR4 signaling pathway, providing a clue to further investigate their roles in the interplay between the parasite and the host.

## Materials and Methods

### Ethics Statement

All animal experiments were assessed and approved by the Animal Ethics Committee of Lanzhou Veterinary Research Institute, Chinese Academy of Agricultural Sciences (LVRI-AX-20180923) and were performed strictly according to the Animal Ethics Procedure and Guidelines of the People's Republic of China.

### Parasites and Sample Preparation

Fertile cysts of *E. granulosus* (G1 genotype) were dissected from the liver of 15 naturally infected sheep slaughtered in an abattoir in the Xinjiang Autonomous Region, China. After aseptic processing, the HF was carefully aspirated with a syringe, mixed, and centrifuged at 300×*g* for 10 min at 4°C to get rid of protoscoleces and cellular debris. The protoscoleces were recovered and purified by repeated natural sedimentation in sterile phosphate-buffered saline (PBS) thrice at 15 min intervals.

### EV Enrichment and Characterization

**Figure 1:**
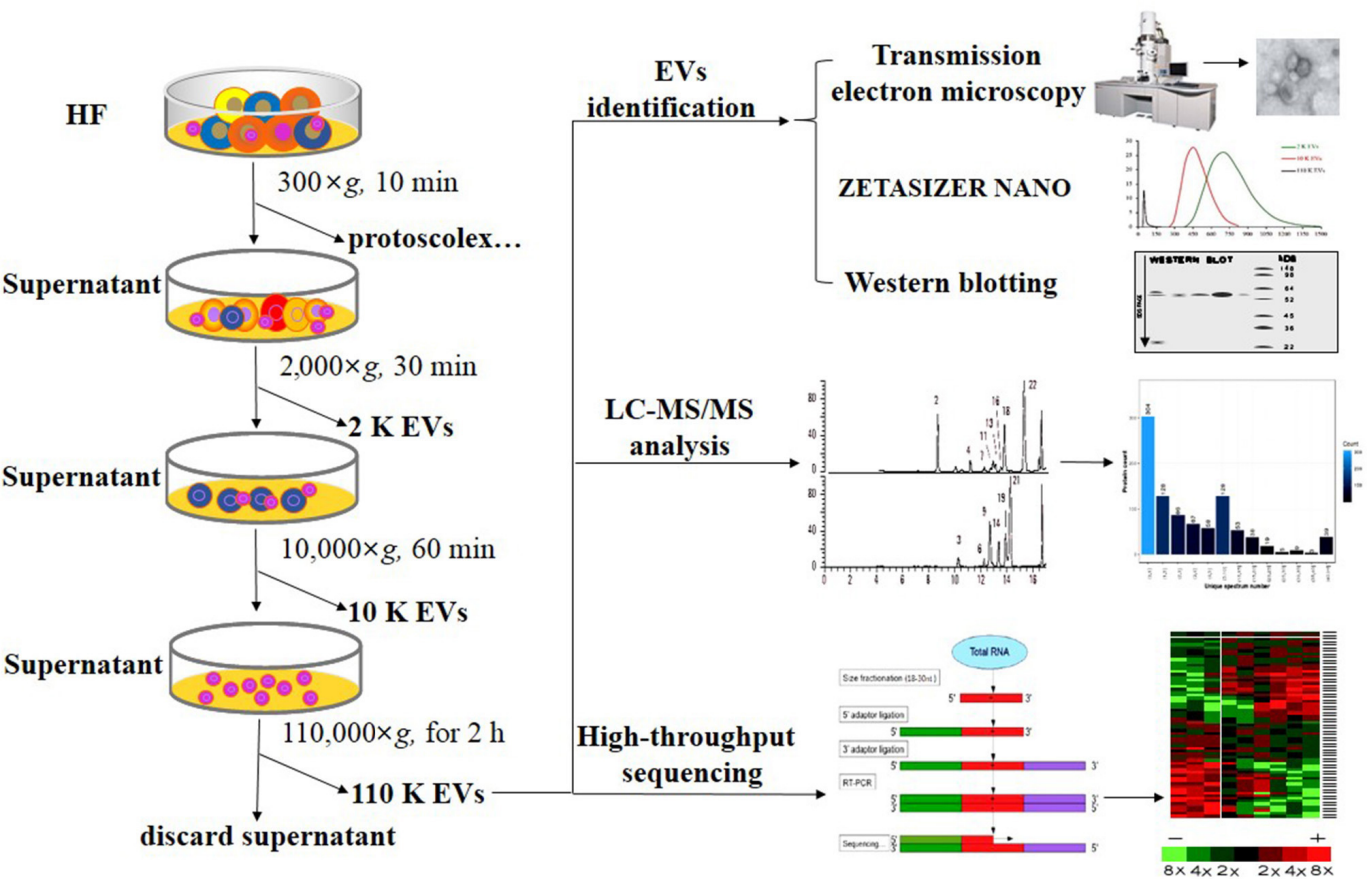
Flow chart of the separation and identification of *E. granulosus* HF EVs. HF, hydatid fluid; EV, extracellular vesicles.

Different EVs were isolated from the protoscolex-free HF as previously reported with some modifications (Figure 1) (22, 27, 32). First, the HF was sequentially centrifuged at 2,000×*g* for 30 min and 10,000×*g* for 60 min at 4°C. Then, the supernatant was filtered using 0.22 μm filters and centrifuged at 110,000 × *g* for 2 h at 4°C. After each centrifugation, the pellets were washed in filtered PBS and recentrifuged using the same centrifugation force. The resultant pellets were named as 2 K, 10 K, and 110 K EVs based on the centrifugation force used. To determine the size and morphology, three different EVs were resuspended in filtered PBS and identified using ZETASIZER NANO (Malvern) and JEM-2010 transmission electron microscopy (TEM, JEOL, Japan) as previously described (25). The results were derived from three independent experiments.

### Proteomic Analysis of EVs Using Mass Spectrometry

The protein cargoes of 2 K, 10 K, and 110 K EVs were defined using LC-MS/MS. To eliminate the host-derived proteins, the data was first mapped against sheep proteins (https://www.ncbi.nlm.nih.gov/genome/?term=sheep), and then, the “clean” data were processed against *E. granulosus* proteins (https://www.genedb.org/#/species/Egranulosus) with parameters as previously described (25). Only proteins with at least two unique peptides were included for the analysis. The identified proteins were subjected to further analysis with Kyoto Encyclopedia of Genes and Genomes (KEGG) pathways and Gene Ontology (GO) terms as previously described (25).

### Protein Preparation and Western Blotting

The soluble crude antigens of *E. granulosus* were prepared by the following protocol. Briefly, 100 mg of *E. granulosus* metacestodes were grounded into powder, followed by the addition of 0.5 mL of PBS containing a proteinase inhibitor cocktail (Sigma, USA). Then, the sample was agitated overnight at 4°C and centrifuged at 12,000×*g* for 15 min at 4°C. Finally, the obtained supernatant was filtered with a 0.22 μm filter and stored at -20°C for later usage.

Total proteins of different EVs were extracted by RIPA Lysis and Extraction Buffer (ThermoFisher Scientific, USA) containing a proteinase inhibitor according to the instructions. Protein concentration was determined using BCA Protein Assay Kit (Solarbio, China). For SDS-PAGE, 30 μg of total proteins per lane were separated by 10% ExpressPlusTM Page Gels (GenScript, China) and transferred to polyvinylidene difluoride (PVDF) membranes. Prior to transfer, the PVDF membrane was activated in methanol for 5 min and then equilibrated in 1× transfer buffer until use. The membranes were blocked with 7.5% defatted milk and then incubated at 4°C overnight with the rabbit anti-14-3-3 antibody and mouse anti-enolase monoclonal antibody (previously prepared in our lab) (33). After being washed in PBST and incubated with the goat anti-rabbit IgG (KPL, Cat No: 5220-0336) or goat anti-mouse IgG (KPL, Cat No: 5220-0341) secondary antibody for 60 min, the membranes were visualized by exposure on x-ray films using Invitrogen Novex ECL HRP Chemiluminescent Substrate Reagent Kit (ThermoFisher Scientific, USA). The results were derived from three independent experiments.

### Small RNA Extraction, High-Throughput Sequencing, and Data Analysis

Small RNA extraction from three different EVs was performed according to the instructions of Invitrogen TRIzol LS Reagent (ThermoFisher Scientific, USA) with some modifications. After homogenization and centrifugation, 1 μL of glycogen (10 mg/mL, Invitrogen) was added into RNA-containing aqueous supernatant. Followed by incubation overnight at -20°C and centrifugation, the pellet was washed with 75% ethanol (prepared using nuclease-free water) and resuspended in nuclease-free water. The concentration and quality of the extracted RNA samples were determined using Bioanalyzer (Agilent, USA).

Small RNA sequencing was carried out using RNA-Seq (Quantification) sequencing (BGI, China). The PAGE gel electrophoresis of extracted total RNA was performed to separate different RNA segments. The small RNA fragments with a size between 18 and 30 nt were isolated and purified. The small RNA was ligated with 3' and 5' adapters and then RT-PCR enrichment was performed by StepOnePlus Real-Time PCR system (ABI, USA). The PCR products were further purified by 10% PAGE and then used for deep sequencing. In the data processing, to obtain clean reads, the repetitive sequences, small degraded mRNA, and the reads with poly A, shorter than 18 nt and without the insert tags, were removed from raw data. The clean reads were mapped to the *E. granulosus* genome (https://parasite.wormbase.org/Echinococcus_granulosus_prjeb121/Info/Index/) by SOAP2 software (BGI). For novel miRNA prediction, the miRDeep2 algorithm and the miReap program were used as previously described (Figure 1) (34, 35).

### Comparative Real-Time PCR

Reverse transcription of miRNAs was performed using 1.0 μg of total RNA by All-in-One miRNA First-strand cDNA Synthesis Kit (GeneCopoeia, USA) according to the protocol of the manufacturer. Before reverse transcription, 3.6 μL of cel-39-5p Spike-In Control (1.6 × 10^8^ copies/μL, Qiagen), a *Caenorhabditis elegans* miRNA, was added as an external reference. The cDNA products were diluted with five volumes of RNase free water, and real-time PCR was immediately performed using an ABI 7500 Thermal Cycler (ThermoFisher Scientific, USA) as follows: 95°C for 10 min, followed by 40 cycles of 95°C for 10 s and 60°C for 1 min. In the experiment, U6 was used as a reference.

For mRNA reverse transcription, RevertAid First Strand cDNA Synthesis Kit (ThermoFisher, USA) was used in strict accordance with the instructions of the manufacturer. About 1.0 μg of total RNA was transcribed with oligo (dT)_18_, and the cDNA products were diluted by adding eight volumes of RNase-free water for later usage. The real-time PCR reactions were set up using All-in-One qPCR Mix (GeneCopoeia, USA) according to the instructions, and 96-well qPCR reaction plates (ThermoFisher Scientific, USA) were used if applicable. To evaluate the relative expression levels of target genes, β-actin was used as a reference in this experiment.

All specific primers used were designed and synthesized (Table 1), and their specificity was confirmed by analysis of the melting curves. The 2^−Δ*ΔCt*^ formula was used to calculate the relative expression levels of the targets. Each sample was tested in triplicate, and the data for the analysis were from three independent experiments.

**Table 1:** The primers used in this study.

**Primer**	**Sequence (5'-3')**
RT primer	GCGAGCACAGAATTAATACGACTCACTATAGG(T)_12_VN^a^
Universal reverse primer	GCGAGCACAGAATTAATACGAC
egr-mir-71	TGAAAGACGATGGTAGTGAGA
egr-let-7	TGAGGTAGTGTTTCGAATGTCT
egr-miR-9	TCTTTGGTTATCTAGCTGTGTG
egr-miR-7	TGGAAGACTGGTGATATGTTGT
egr-miR-2a	AATCACAGCCCTGCTTGGAACC
egr-miR-219	TGATTGTCCATTCGCATTTCTTG
egr-miR-190	AGATATGTTTGGGTTACTTGGTG

a *"V” stands for A, G, or C; “N” stands for A, T, G, or C*.

### Isolation of Sheep PBMCs

About 10 mL of blood was collected from each of the three healthy sheep, and PBMCs were separately isolated by polysucrose-diammonia density gradient centrifugation according to the instructions of the manufacturer (TMD Science, China) (36). The three PBMC samples were washed in three volumes of pre-chilled PBS, and cell viability was checked using trypan blue.

### Uptake of 110 K EVs by PBMCs and *E. granulosus* Protoscolex

Purified 110 K EVs were labeled with PKH26 (Sigma, USA) according to the instructions of the manufacturer. PBMCs were cultured in RPMI-1640 medium supplemented with 10% exosome-free FBS (Gibco, USA). About 3 × 10^6^ cells were seeded into each well in 6-well plates (Corning, USA) and cultured with 100 μg of the labeled EVs per well. About 0, 4, 8, 12, and 24 h after seeding, PBMCs were collected, fixed by 4% formaldehyde, and then washed by PBS. PBMCs were labeled with ActinGreen (Invitrogen, USA) and DAPI (Sigma, USA). The internalization was assessed using laser confocal microscopy (×100) (Leica, Germany) and flow cytometry (Merck, USA), and the internalization rate at different time points was evaluated thrice. For the flow cytometry, 100 μg labeled 110 K EVs were incubated with 3 × 10^6^ PBMCs, and a total of ~10,000 cells were analyzed by Guava easyCyte 6-2L (Merck) at different time points. Furthermore, to detect whether 110 K EVs can be internalized by protoscolex, 100 μg of the labeled EVs was added into 1 × 10^4^ parasites and observed under fluorescent microscopy (Leica, Germany) at 24 h and 48 h after treatment. In parallel, PBS was processed similar to that of 110 K EVs and used as a control. The results were derived from three independent experiments.

### Enzyme-Linked Immunosorbent Assay (ELISA)

The levels of tumor necrosis factor-α (TNF–α) in the supernatant of sheep PBMC culture were detected using a Double Antibody Sandwich ELISA Kit (JL, China) according to the instructions of the manufacturer. Briefly, 100 μL of the supernatant and standard were added into each well and incubated for 2 h at 37°C. After wash, 100 μL of biotin-antibody (1×) was added into each well and incubated for 1 h at 37°C, followed by wash and addition of HRP-avidin. After addition of TMB substrate and stop solution, the optical density at 570 nm and 450 nm was recorded using a microplate reader (Bio-Rad, USA). Each sample and standard was set in triplicate.

### Statistical Analysis

GraphPad Prism 5 was used for data analysis, and statistical significance was analyzed using a two-tailed unpaired *t*-test or ANOVA. If the *p* < 0.05, it was considered significantly different.

## Results

### Identification of Different HF EVs

**Figure 2:**
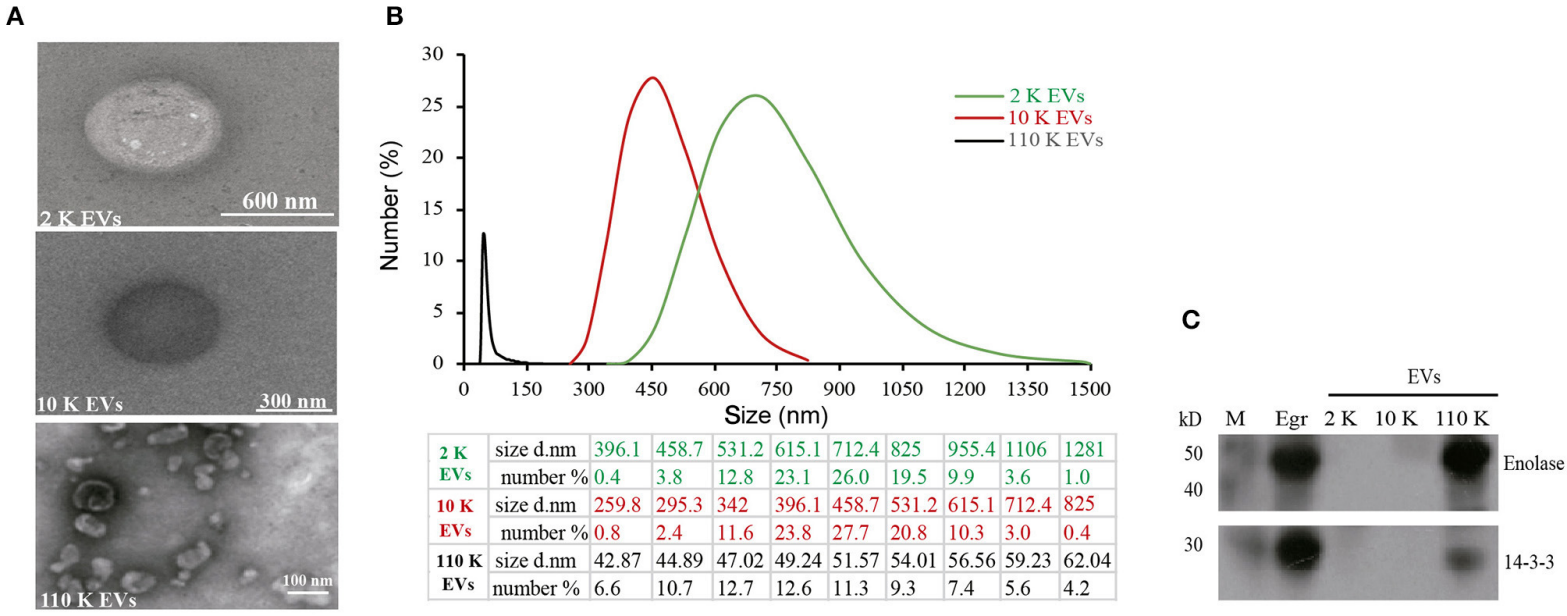
Identification of different *E. granulosus* HF EVs. **(A)** Transmission electron microscopy (TEM) of different HF EVs. **(B)** The size distribution of different HF EVs. **(C)** Western blotting analysis of enolase and 14-3-3 in different HF EVs. HF, hydatid fluid; EV, extracellular vesicles; M, marker.

Consistent with the size distribution, the results of TEM showed the spherical EVs with different sizes. Of them, 2 K EVs mainly ranged from 531 to 955 nm, 10 K EVs mainly ranged from 342 to 615 nm, and 110 K EVs mainly ranged from 45 to 54 nm (Figures 2A,B). Furthermore, both 14-3-3 and enolase, two exosome biomarkers, were only present in 110 K EVs but not in 2 K and 10 K HF EVs (Figure 2C).

### Protein Cargoes of Different HF EVs

**Figure 3:**
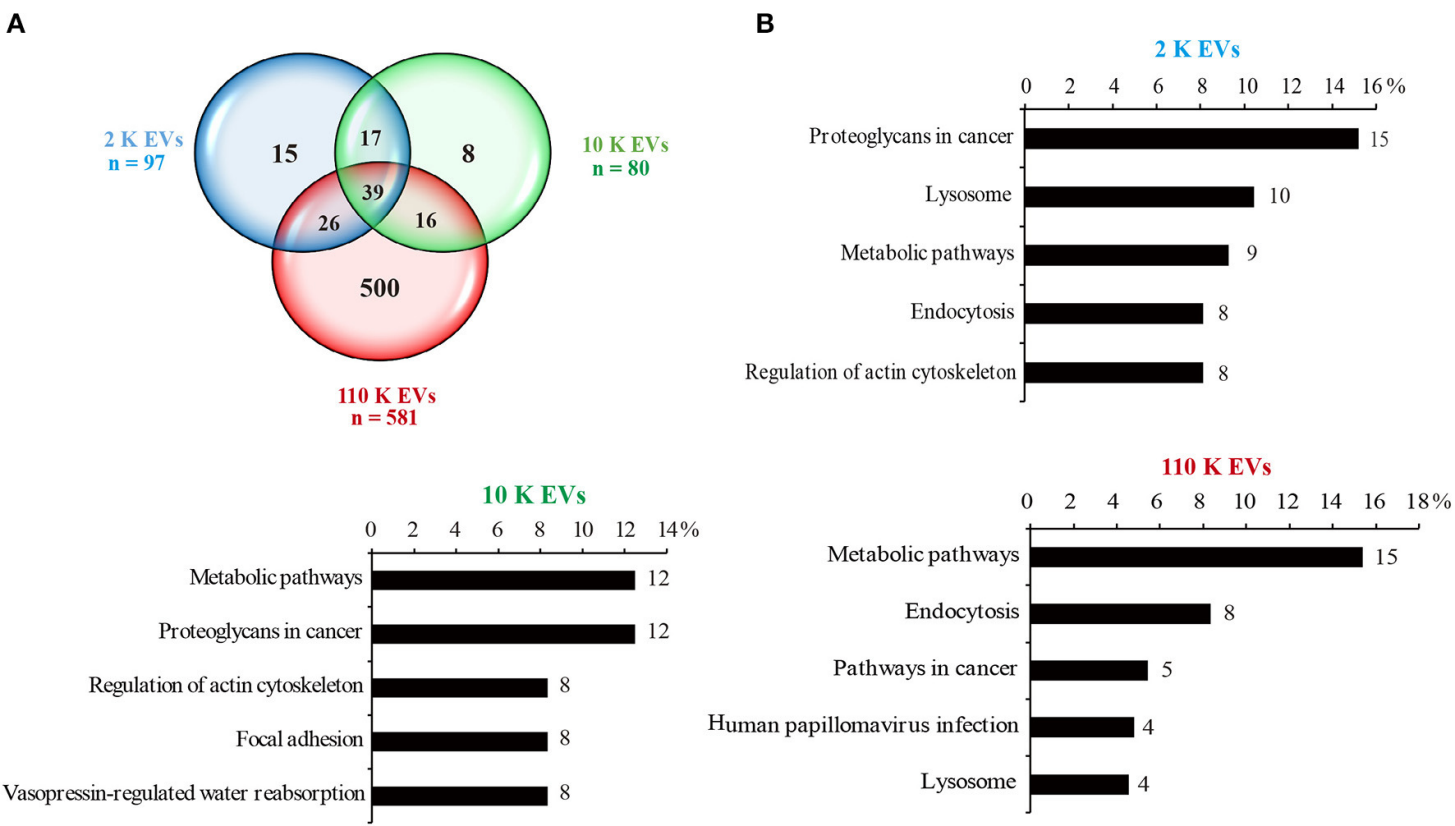
Protein cargoes of different *E. granulosus* HF EVs. **(A)** Venn diagram of the protein cargoes in different HF EVs. **(B)** KEGG analysis of different HF EVs. HF, hydatid fluid; EV, extracellular vesicles; KEGG, Kyoto Encyclopedia of Genes and Genomes.

In total, 97, 80, and 581 proteins were detected from 2 K, 10 K, and 110 K EVs, respectively (Supplementary Tables 1–3). Of them, 15, 8, and 500 proteins were exclusively present in 2 K, 10 K, and 110 K EVs, respectively, and only 39 were commonly shared (Figure 3A). KEGG analysis revealed that the proteins of 10 K and 110 K EVs were predominantly involved in metabolic pathways, up to ~12 and 15% of annotated proteins, respectively (Figure 3B). Moreover, the proteins involved in the lysosome and endocytosis were also abundant in 2 K and 110 K EVs (Figure 3B).

### miRNA Profiles of Different HF EVs

**Figure 4:**
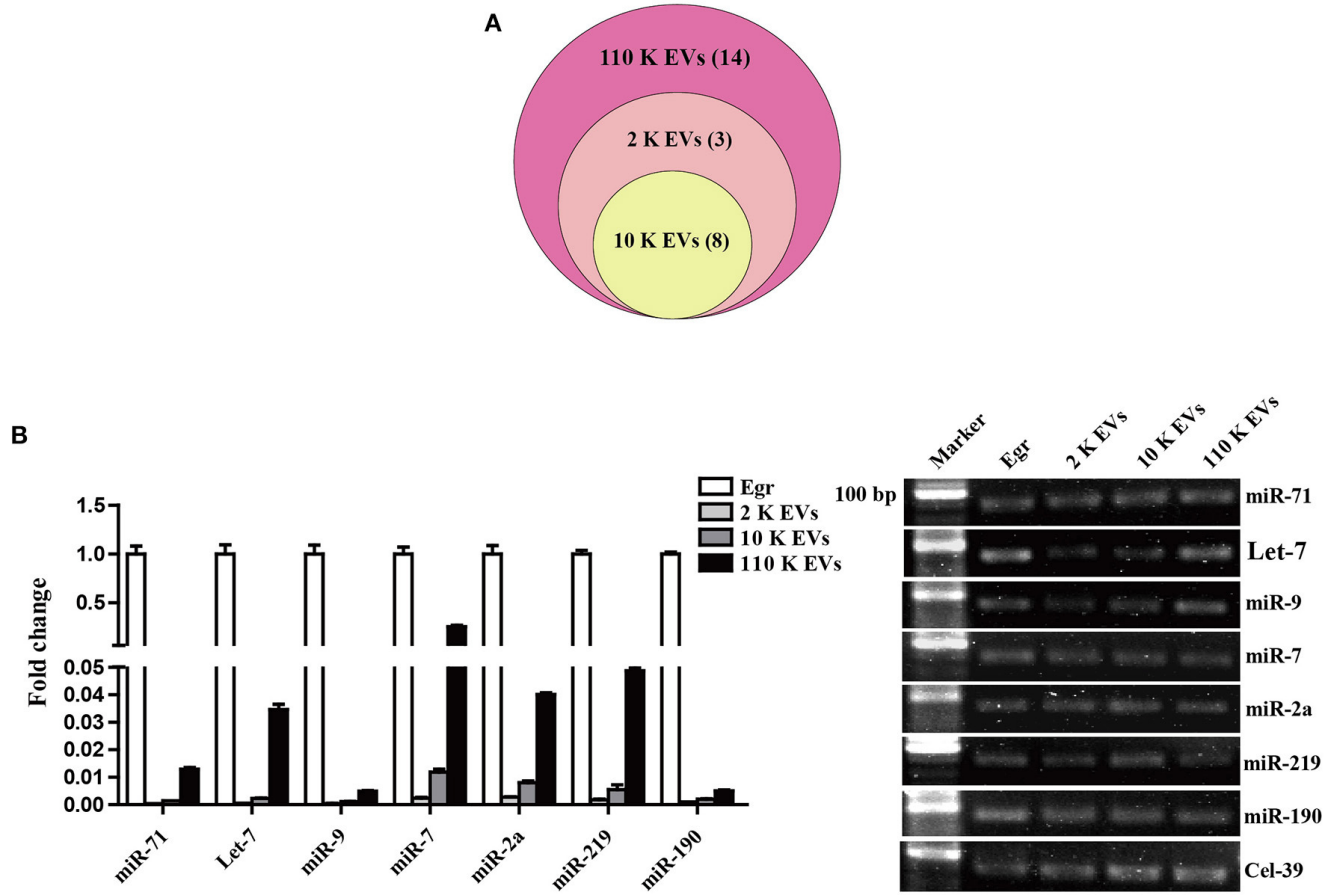
Identification of relevant miRNAs in different *E. granulosus* HF EVs. **(A)** Venn diagram of miRNA cargoes in different HF EVs. **(B)** Validation of the top seven known miRNAs by real-time PCR. *E. granulosus* protoscoleces (Egr) were used as a control. 20 bp DNA ladder markers were shown on the left. HF, hydatid fluid; EV, extracellular vesicles.

The high-throughput sequencing results showed that the size of miRNA cargoes in three different HF EVs ranged from 19 to 23 nt (Supplementary Table 4). In total, 11, 8, and 25 miRNAs were detected from 2 K, 10 K, and 110 K EVs, respectively. Of them, eight miRNAs were commonly shared (Figure 4A). In 110 K EVs, egr-miR-71 had the highest abundance, followed by egr-let-7 and egr-miR-9. However, some miRNAs abundant in 110 K EVs, such as egr-miR-7, egr-miR-190, and egr-miR-277, were undetectable in 2 K or/and 10 K EVs (Supplementary Table 4).

To validate the sequencing data, the relative abundance of the top seven known miRNAs in HF EVs, namely egr-miR-71, egr-let-7, egr-miR-9, egr-miR-7, egr-miR-2a, egr-miR-219, and egr-miR-190, was assessed by real-time PCR. In general, the real-time PCR results were consistent with the sequencing data and confirmed that all miRNAs were the most abundant in 110 K EVs compared with 2 K and 10 K EVs (Figure 4B, Supplementary Table 4). However, except for egr-miR-7, the other six miRNAs were much less abundant in all the three types of HF EVs compared with parasites themselves (Figure 4B).

### Regulatory Effects of 110 K HF EVs on Sheep PBMCs

**Figure 5:**
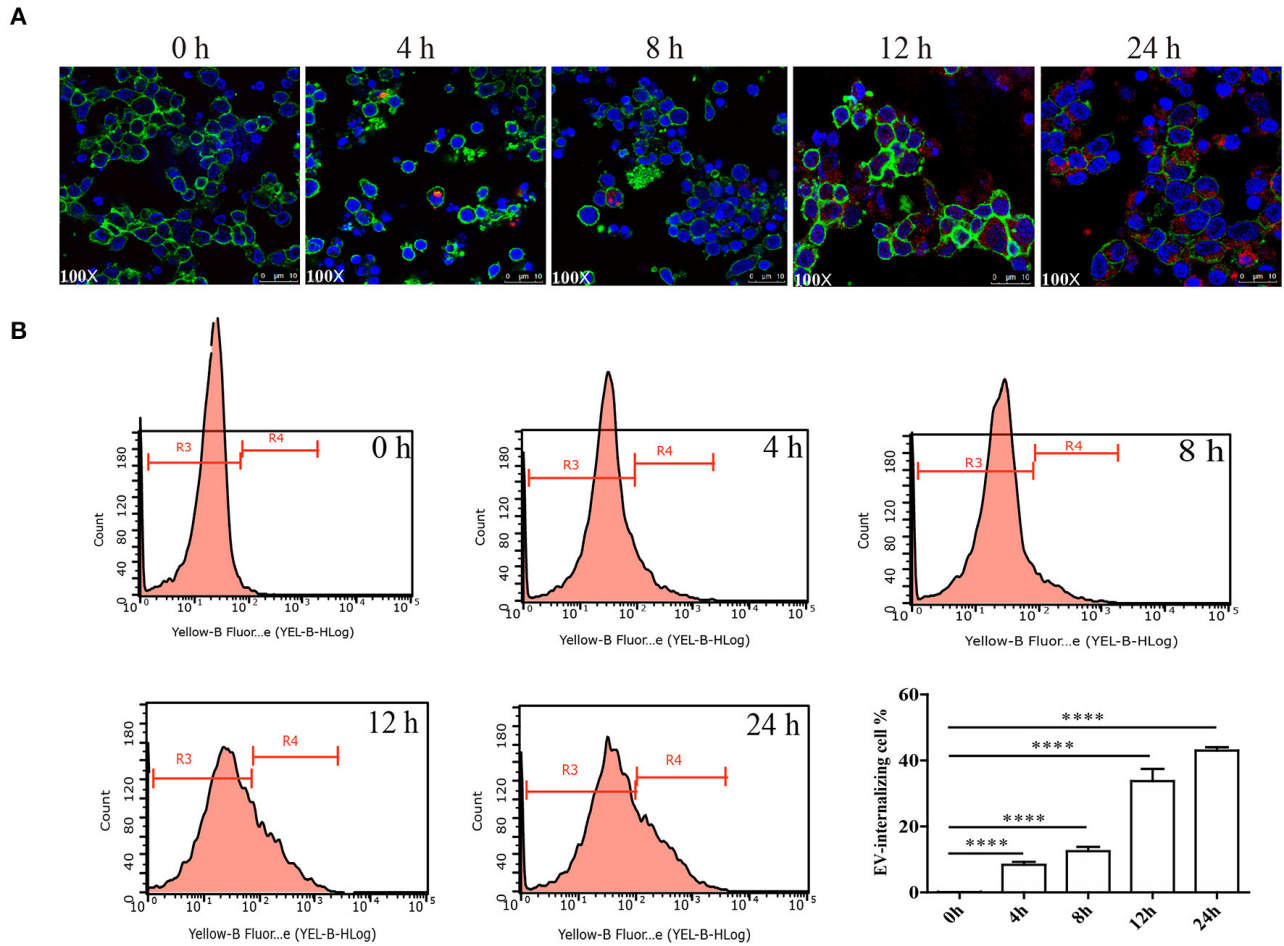
Internalization of *E. granulosus* 110 K HF EVs by sheep PBMCs. **(A)** Internalization of 110 K HF EVs by PBMCs. 110 K HF EVs were labeled with PKH26, PBMC was labeled with ActinGreen, and the nucleus was stained by DAPI. **(B)** Internalization analysis *via* flow cytometry. ****p < 0.0001. The R3 region represents the proportion of non-EV-internalizing PBMCs, while the R4 represents the proportion of EV-internalizing ones. HF, hydatid fluid; EV, extracellular vesicles; PBMCs, peripheral blood mononuclear cells.

It was confirmed that the 110 K HF EVs were internalized by sheep PBMCs *in vitro* in a time-dependent manner (Figures 5A,B). PBMCs incubated with PKH26-labeled 110 K HF EVs had bright red fluorescent signals at 12 and 24 h after incubation (Figure 5A), and the internalization rate was over 40% at 24 h after incubation (Figure 5B). To determine whether the 110 K HF EVs are internalized by *E. granulosus* protoscolex, an internalization assay was also performed, but no fluorescent signals were observed in parasites (data not shown).

**Figure 6:**
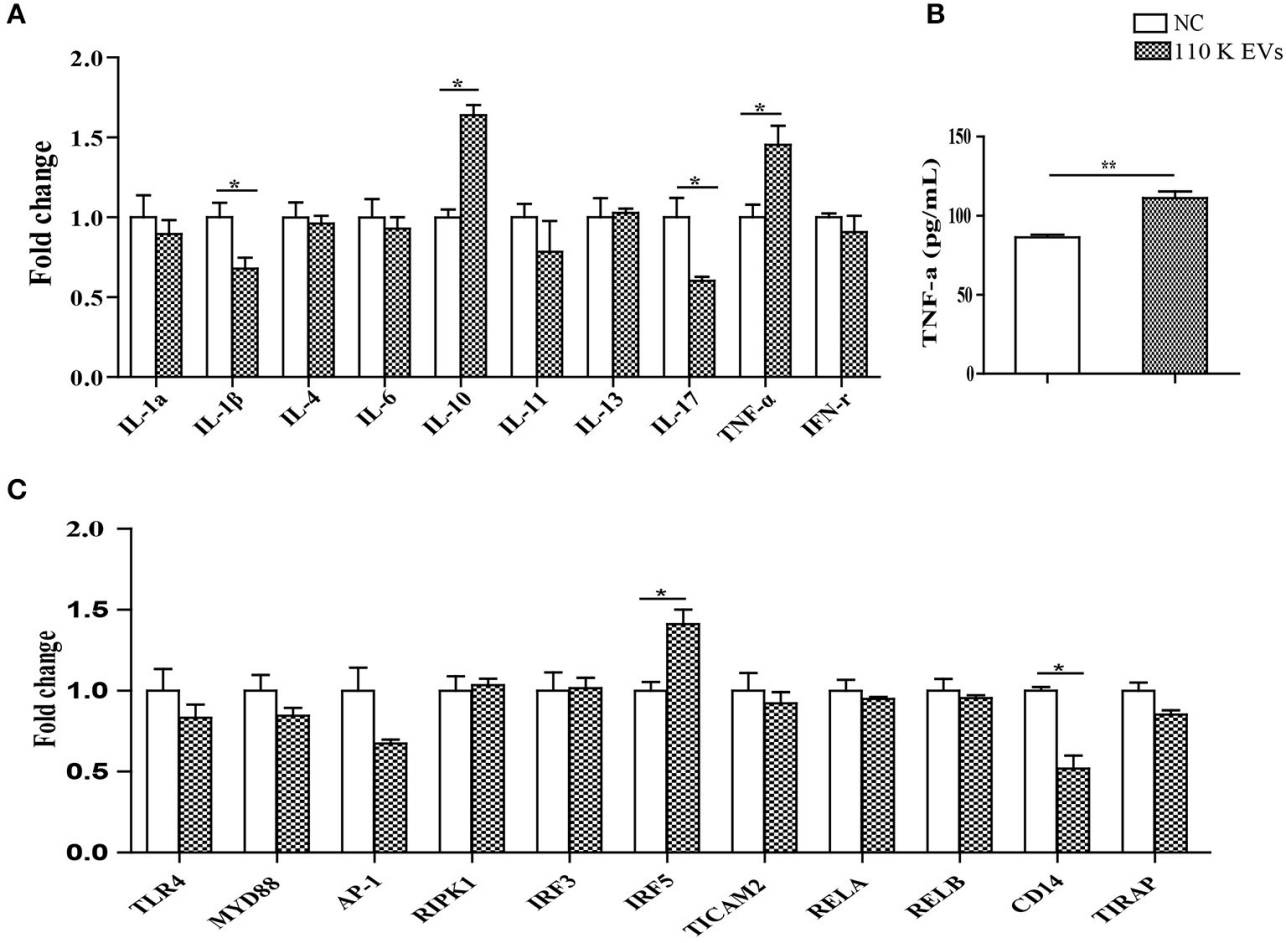
Regulatory effects of 110 K HF EVs on the expression of cytokines and core components in the LPS/TLR4 signaling pathway in sheep PBMCs. **(A)** qPCR analysis of the expression of cytokines after treatment with 110 K HF EVs. Data are expressed as mean ± s.e.m. Data for the analyses are from three independent experiments, *p < 0.05. **(B)** Sandwich ELISA analysis of the expression of TNF-α after treatment with 110 K HF EVs. Data are expressed as mean ± s.e.m. Data for the analyses are from three independent experiments, **p < 0.01. **(C)** qPCR analysis of the expression of the core components in the LPS/TLR4 pathway after treatment with 110 K HF EVs. Data are expressed as mean ± s.e.m. Data for the analyses are from three independent experiments, *p < 0.05. HF, hydatid fluid; EV, extracellular vesicles; PBMCs, peripheral blood mononuclear cells; NC, negative control.

It has been demonstrated that EVs can transfer their own biological factors and effector molecules from parasites to hosts, thereby regulating immune responses (26). To further determine whether the 110 K HF EVs also have immunomodulatory effects on host cells, such as PBMCs, real-time PCR experiments were carried out. The results showed that 110 K EVs induced the significant upregulation of IL-10 and TNF-α (*p* < 0.05) in treated sheep PBMCs, with IL-17 and IL-1β being significantly downregulated 24 h after treatment (*p* < 0.05, Figure 6A). Consistently, the result of ELISA verified that TNF-α was significantly increased in the treated PBMCs (*p* < 0.01, Figure 6B).

For the core components in the TLR4/LPS pathway, only IRF5 and CD14 were significantly upregulated and downregulated (*p* < 0.05), respectively, and the rest remained constant after treatment (Figure 6C).

## Discussion

Cystic echinococcosis remains a considerable health crisis in many regions across the world, including western China, Central Asia, South America, and eastern Africa (2), partially due to limited understanding of the pathogenesis (37). In this study, we isolated and identified three types of EVs from *E. granulosus* HF for the first time and found that the diameter of 110 K EVs was from 40 to 150 nm, which was similar to the exosome-like EVs from *Trypanosoma brucei* (38), *Acanthamoeba castellanii* (39), and *Brugia malayi* (40). In accordance to this finding, both enolase and 14-3-3, which are normally used as exosomal biomarkers (25, 32), were detected only in 110 K EVs. Simultaneously, we thoroughly characterized the miRNAs and proteins of different HF EVs and found that some proteins, such as annexin and tetraspanin, were commonly enriched.

Gene Ontology and KEGG analysis showed many proteins being involved in the cellular process and the metabolic process, which was consistent with the results of the proteomic analysis on exosomes derived from the sera of patients infected with *E. granulosus* and on excretory/secretory (E/S) products of *E. granulosus* metacestode (14, 41). A number of studies have shown that E/S products have overlapped components with EVs, which are supposed to be a candidate as a biological predictor and biomarker (2, 14, 25, 42). It is envisaged that the full characterization of the HF EVs in the study shed light on the identification of reliable diagnostic biomarkers, which will be useful for management of patients. On the other hand, it was found that 110 K HF EVs had a complex protein composition, especially ones that induce immune protection (43, 44), suggesting a potential for developing a vaccine. For instance, it was reported that the 14-3-3 protein provided 97% protection against *E. multilocularis* infection in rodents (45). Therefore, the current data are valuable for developing anti-echinococcosis vaccines.

miRNAs can be transported from parasites to host cells and modulate innate immunity by exosome-like vesicles (30). We herein showed that *E. granulosus* HF EVs contained a specific set of miRNAs, and egr-miR-71 was absolutely predominant in the 110 K EVs. Similarly, miR-71 was also abundant in the exosomes secreted by other parasites, such as *Taenia asiatica, Taenia hydatigena, Schistosoma mansoni*, and *Schistosoma japonicum* (32, 35, 46, 47). However, the role of the exosomal miR-71 remains elusive. Recently, *E. multilocularis* miR-71 was shown to suppress nemo-like kinase, and its overexpression inhibited the production of nitric oxide (NO) by macrophages, suggesting a role in the regulation of immune responses (48, 49). In addition, egr-miR-71 was present in the plasma of patients at the early stage of infection and had a higher level than that of egr-let-7, which suggested that egr-miR-71 might serve as a novel diagnostic biomarker and might monitor hydatidosis at the early stage (50). In future studies, it is imperative to decipher the role of 110 K EV-derived egr-miR-71 during *E. granulosus* infection.

The secretion of EVs has profound biological effects, in that the encapsulated proteins, lipids, and nucleic acids are biologically functional when EVs are internalized by adjacent and distant cells (51). EVs facilitate the direct extracellular transfer of active molecules *in vitro* and *in vivo* (52). 110 K HF EVs were *in vitro* internalized by sheep PBMCs, suggesting a role in signal transduction between the parasite and the host that regulates the process of *E. granulosus* infection. In accordance with this idea, internalized 110 K HF EVs induced significant changes in the expression of four cytokines, including IL-1β, IL-10, IL-17, and TNF-α. Similarly, two key components (IRF5 and CD14) in the LPS/TLR4 pathway were also ectopically expressed. It is well-known that IRF5 is involved in the production of inflammatory cytokines such as IL-6 and TNF-α. In the study, IRF5 and TNF-α was significantly upregulated, while IL-6 was still unchanged. This may result from the presence of plenty of different immune mediators in 110 K EVs that synthetically regulate the synthesis of IL-6 *via* multiple pathways. Although the exact mechanisms behind are unclear, the data suggest a modulatory role of 110 K HF EVs, providing a new direction to investigate the biological functions of 110 K EVs in the parasite–host interplay.

In future, investigating immune responses to 110 K HF EVs in sheep should be of great interest. This will be helpful for us to understand their role in the pathogenesis. In addition, it will also be interesting to pinpoint the role of HF EVs in human patients to study the process of infection and immunity.

## Data Availability Statement

The datasets generated in this study have been deposited into the BioProject database (accession number: PRJNA674015).

## Ethics Statement

The animal study was reviewed and approved by Animal Ethics Committee of Lanzhou Veterinary Research Institute, Chinese Academy of Agricultural Sciences, China.

## Author Contributions

YZhe conceived the study. JY, JW, YF, LY, and YL conducted the experiments. JY, JW, XG, YZha, XW, and YS analyzed the data. JY, YZhe, WCC, and YS wrote the paper. All authors contributed to the article and approved the submitted version.

## Conflict of Interest

The authors declare that the research was conducted in the absence of any commercial or financial relationships that could be construed as a potential conflict of interest.

## Abbreviations

EVs: extracellular vesicles
HF: hydatid fluid
PBMCs: peripheral blood mononuclear cells
CE: cystic echinococcosis
AE: alveolar echinococcosis
DALYs: disability-adjusted life years
IL: interleukin
LC-MS/MS: liquid chromatography-coupled tandem mass spectrometry
KEGG: Kyoto Encyclopedia of Genes and Genomes
GO: Gene Ontology
TNF-α: tumor necrosis factor-α
NO: nitric oxide.
