# Supplementary material for: Identification of Different Extracellular Vesicles in the Hydatid Fluid of Echinococcus granulosus and Immunomodulatory Effects of 110 K EVs on Sheep PBMCs
Source: Front Immunol. 2021 Feb 23;12:602717. doi: 10.3389/fimmu.2021.602717 (PMC7940240; doi:10.3389/fimmu.2021.602717)
Supplement: Supplementary file 1 [file Table_1.DOCX]

Table S1 Protein cargoes of *E. granulosus* 2 K HF EVs

| **Protein** | | **Protein ID** | **Size (kDa)** | | **Unique Peptide** | | **Unique Spectra** | |
| --- | --- | --- | --- | --- | --- | --- | --- | --- |
| Expressed Conserved Protein | EgrG_001061900 | | | 74 | | 25 | 27 |  |
| Annexin | EgrG_000244000 | | | 37 | | 17 | 20 |  |
| Tetraspanin | EgrG_000355700 | | | 31 | | 11 | 18 |  |
| Citrate Synthase | EgrG_001028500 | | | 51 | | 11 | 12 |  |
| Dynein Light Chain | EgrG_000990800 | | | 11 | | 10 | 14 |  |
| Myoferlin | EgrG_000825200 | | | 230 | | 10 | 10 |  |
| Dynein Light Chain 1, Cytoplasmic | EgrG_000991200 | | | 12 | | 9 | 17 |  |
| Glipr1-Like Protein | EGR_09283 | | | 27 | | 9 | 14 |  |
| Annexin | EgrG_000041200 | | | 39 | | 9 | 10 |  |
| Tsp1 | EgrG_000355800 | | | 29 | | 8 | 10 |  |
| Annexin | EgrG_000243600 | | | 35 | | 8 | 10 |  |
| Guanylate Cyclase | EgrG_000928050 | | | 101 | | 8 | 9 |  |
| Tetraspanin | EgrG_001077100 | | | 24 | | 7 | 9 |  |
| Actin Cytoplasmic A3 | EgrG_000406900 | | | 42 | | 7 | 8 |  |
| Major Egg Antigen | EgrG_000236500 | | | 37 | | 6 | 9 |  |
| Anoctamin | EgrG_000175600 | | | 117 | | 6 | 7 |  |
| Uncharacterized Protein | EGR_10334 | | | 30 | | 6 | 7 |  |
| Annexin | EgrG_000243700 | | | 34 | | 6 | 6 |  |
| Estrogen Regulated Protein Ep45 | EgrG_000824100 | | | 50 | | 6 | 9 |  |
| Expressed Conserved Protein | EgrG_000806200 | | | 27 | | 6 | 7 |  |
| Expressed Protein | EgrG_001110600 | | | 113 | | 6 | 7 |  |
| Tetraspanin | EgrG_000354700 | | | 27 | | 5 | 6 |  |
| Dynein Light Chain | EgrG_000991000 | | | 12 | | 5 | 9 |  |
| Polyubiquitin | EgrG_000516500 | | | 15 | | 5 | 7 |  |
| Dynein Light Chain | EgrG_000182400 | | | 19 | | 5 | 7 |  |
| Dynein Light Chain | EgrG_000990900 | | | 11 | | 5 | 9 |  |
| Peptidase Inhibitor | EGR_04630 | | | 27 | | 5 | 6 |  |
| Antigen B 1/1 (Fragment) | EgrG_000381100 | | | 7 | | 5 | 6 |  |
| Syndecan Binding Protein Syntenin | EgrG_000453900 | | | 31 | | 5 | 6 |  |
| Annexin | EgrG_000193700 | | | 39 | | 5 | 6 |  |
| Phosphatidate Phosphatase | EgrG_000635000 | | | 29 | | 5 | 5 |  |
| Profilin | EgrG_000122100 | | | 13 | | 4 | 5 |  |
| Uncharacterized Protein | EGR_08255 | | | 38 | | 4 | 5 |  |
| Actin Cytoplasmic Type 5 | EgrG_000190400 | | | 40 | | 4 | 4 |  |
| Expressed Conserved Protein | EgrG_000120300 | | | 39 | | 4 | 5 |  |
| Atp Binding Cassette Subfamily B Mdr:Tap | EgrG_000901000 | | | 132 | | 4 | 4 |  |
| Elongation Factor 1-Alpha | EgrG_000982200 | | | 50 | | 4 | 4 |  |
| Serine Protease Inhibitor | EgrG_001193200 | | | 39 | | 4 | 4 |  |
| Thioredoxin Glutathione Reductase | EgrG_000222300 | | | 58 | | 4 | 4 |  |
| Uncharacterized Protein | EGR_11318 | | | 14 | | 3 | 4 |  |
| Dynein Light Chain Type 1 2 | EgrG_000991300 | | | 10 | | 3 | 6 |  |
| Expressed Protein | EgrG_000724500 | | | 9 | | 3 | 3 |  |
| Tetraspanin | EgrG_000834300 | | | 25 | | 3 | 3 |  |
| Glycoside Hydrolase Subgroup Catalytic Core | EgrG_000059500 | | | 58 | | 3 | 3 |  |
| Phospholipid Scramblase | EgrG_000624400 | | | 34 | | 3 | 3 |  |
| Expressed Protein | EgrG_000260400 | | | 35 | | 3 | 3 |  |
| Expressed Conserved Protein | EgrG_001085900 | | | 23 | | 3 | 5 |  |
| Annexin | EgrG_000041300 | | | 37 | | 3 | 4 |  |
| Expressed Protein | EgrG_000513800 | | | 33 | | 3 | 3 |  |
| Histone H4 | EgrG_000323100 | | | 11 | | 3 | 4 |  |
| Transporter | EgrG_000220200 | | | 87 | | 3 | 3 |  |
| Neutral Amino Acid Transporter A | EgrG_001168200 | | | 33 | | 3 | 3 |  |
| 14-3-3 Protein | EgrG_001192500 | | | 28 | | 3 | 3 |  |
| Collagen Alpha 1V Chain | EgrG_000144300 | | | 177 | | 3 | 3 |  |
| Uncharacterized Protein | EGR_05598 | | | 185 | | 3 | 3 |  |
| Expressed Conserved Protein | EgrG_000681600 | | | 8 | | 2 | 3 |  |
| Uncharacterized Protein | EGR_03661 | | | 8 | | 2 | 2 |  |
| Tapeworm Specific Antigen B | EgrG_000381400 | | | 10 | | 2 | 2 |  |
| Expressed Conserved Protein | EgrG_000968300 | | | 28 | | 2 | 2 |  |
| Tctex1 Domain-Containing Protein | EGR_07068 | | | 50 | | 2 | 2 |  |
| Vesicle Associated Membrane Protein | EgrG_000494400 | | | 31 | | 2 | 2 |  |
| Tetraspanin | EgrG_001077400 | | | 24 | | 2 | 2 |  |
| Mitochondrial Atp Synthase Subunit 9 | EgrG_001064800 | | | 13 | | 2 | 2 |  |
| Shc Transforming Protein 3 | EgrG_001062000 | | | 48 | | 2 | 2 |  |
| Dynein Light Chain | EgrG_000941000 | | | 22 | | 2 | 3 |  |
| Tetraspanin | EgrG_000833400 | | | 28 | | 2 | 3 |  |
| Expressed Conserved Protein | EgrG_000682000 | | | 24 | | 2 | 2 |  |
| Annexin | EgrG_000330300 | | | 48 | | 2 | 2 |  |
| Glutathione S-Transferase | EgrG_000538900 | | | 25 | | 2 | 3 |  |
| Ras Protein Rabf2B | EgrG_001004250 | | | 20 | | 2 | 2 |  |
| Tetraspanin | EgrG_001021300 | | | 24 | | 2 | 2 |  |
| Rab | EgrG_000349500 | | | 24 | | 2 | 2 |  |
| Thioredoxin Fold | EgrG_000666500 | | | 14 | | 2 | 2 |  |
| T Cell Immunomodulatory Protein | EgrG_000440000 | | | 64 | | 2 | 2 |  |
| Metacestode Specific Membrane Protein 1 | EgrG_000353400 | | | 24 | | 2 | 2 |  |
| Zinc Transporter Zip8 | EgrG_000946600 | | | 60 | | 2 | 2 |  |
| Charged Multivesicular Body Protein 2A | EgrG_000899000 | | | 29 | | 2 | 2 |  |
| Ubiquitin Family Member Ubq 1 | EgrG_001086700 | | | 39 | | 2 | 2 |  |
| G1Y162 Protein | EgrG_000515900 | | | 17 | | 2 | 2 |  |
| Amino Acid Transporter | EgrG_000469500 | | | 53 | | 2 | 2 |  |
| Ribosomal Protein S3 | EgrG_001167300 | | | 26 | | 2 | 2 |  |
| Acetylcholinesterase | EGR_07877 | | | 79 | | 2 | 2 |  |
| Uncharacterized Protein | EgrG_000432200 | | | 270 | | 2 | 2 |  |
| Basement Membrane Specific Heparan Sulfate | EgrG_000701800 | | | 96 | | 2 | 2 |  |
| Potassium Channel Subfamily K 8 | EgrG_000885600 | | | 62 | | 2 | 2 |  |
| Expressed Conserved Protein | EgrG_001018600 | | | 62 | | 2 | 2 |  |
| Prosaposin A Preproprotein | EgrG_000733100 | | | 114 | | 2 | 2 |  |
| Gelsolin | EgrG_000882300 | | | 42 | | 2 | 2 |  |
| Expressed Conserved Protein | EgrG_000756700 | | | 57 | | 2 | 2 |  |
| Oxalate:Formate Antiporter | EgrG_000661800 | | | 53 | | 2 | 2 |  |
| Lipid Transport Protein N Terminal | EgrG_000684200 | | | 344 | | 2 | 2 |  |
| Pfam-B_1491 And Pfam-B_5139 Domain Containing Protein | EgrG_000773000 | | | 16 | | 2 | 2 |  |
| Chloride Intracellular Channel Protein 5 | EGR_04447 | | | 30 | | 2 | 2 |  |
| Expressed Conserved Protein | EgrG_000120600 | | | 15 | | 2 | 2 |  |
| Placenta Specific Gene 8 Protein | EgrG_000703600 | | | 16 | | 2 | 2 |  |
| V-Type Proton Atpase Proteolipid Subunit | EgrG_001052800 | | | 21 | | 2 | 2 |  |
| Expressed Conserved Protein | EgrG_000042100 | | | 13 | | 2 | 2 |  |
