# Supplementary material for: Identification of Different Extracellular Vesicles in the Hydatid Fluid of Echinococcus granulosus and Immunomodulatory Effects of 110 K EVs on Sheep PBMCs
Source: Front Immunol. 2021 Feb 23;12:602717. doi: 10.3389/fimmu.2021.602717 (PMC7940240; doi:10.3389/fimmu.2021.602717)
Supplement: Supplementary file 2 [file Table_2.DOCX]

Table S2 Protein cargoes of *E. granulosu**s* 10 K HF EVs

| **Protein** | **Protein ID** | **Size (kDa)** | **Unique Peptide** | **Unique Spectra** |
| --- | --- | --- | --- | --- |
| Expressed Conserved Protein | EgrG_001061900 | 74 | 28 | 49 |
| Lipid Transport Protein N Terminal | EgrG_000684200 | 344 | 16 | 16 |
| Estrogen Regulated Protein Ep45 | EgrG_000824100 | 50 | 12 | 14 |
| Major Egg Antigen | EgrG_000236500 | 37 | 11 | 15 |
| Ag5 | EgrG_000184900 | 55 | 8 | 10 |
| Dynein Light Chain 1, Cytoplasmic | EgrG_000991200 | 12 | 8 | 10 |
| Tetraspanin | EgrG_000355700 | 31 | 7 | 9 |
| Expressed Conserved Protein | EgrG_001018600 | 62 | 7 | 7 |
| ATP Binding Cassette Subfamily B Mdr:Tap | EgrG_000901000 | 132 | 7 | 7 |
| Actin Cytoplasmic Type 5 | EgrG_000190400 | 40 | 6 | 7 |
| Tetraspanin | EgrG_001077100 | 24 | 6 | 6 |
| Expressed Conserved Protein | EgrG_000806200 | 27 | 5 | 6 |
| Polyubiquitin | EgrG_000516500 | 15 | 5 | 5 |
| Heat Shock 70 Protein 4 | EgrG_001085400 | 71 | 5 | 6 |
| Annexin | EgrG_000193700 | 39 | 5 | 5 |
| Annexin | EgrG_000243600 | 35 | 5 | 6 |
| Phosphoenolpyruvate Carboxykinase | EgrG_000292700 | 71 | 5 | 5 |
| Annexin | EgrG_000244000 | 37 | 5 | 5 |
| Annexin | EgrG_000041200 | 39 | 5 | 5 |
| Tsp1 | EgrG_000355800 | 29 | 4 | 5 |
| Dynein Light Chain | EgrG_000991000 | 12 | 4 | 6 |
| Expressed Conserved Protein | EgrG_000120300 | 39 | 4 | 4 |
| Shc Transforming Protein 3 | EgrG_001062000 | 48 | 4 | 4 |
| Anoctamin | EgrG_000175600 | 117 | 4 | 4 |
| Dynein Light Chain | EgrG_000990800 | 11 | 4 | 5 |
| Actin Cytoplasmic A3 | EgrG_000406900 | 42 | 4 | 4 |
| Uncharacterized Protein | EgrG_001115400 | 95 | 4 | 4 |
| Thioredoxin Peroxidase | EgrG_000791700 | 21 | 4 | 5 |
| Uncharacterized Protein | EGR_10334 | 30 | 4 | 4 |
| Elongation Factor 1-Alpha | EgrG_000982200 | 50 | 4 | 4 |
| Guanylate Cyclase | EgrG_000928050 | 101 | 4 | 4 |
| Uncharacterized Protein | EgrG_000432200 | 270 | 4 | 5 |
| Egf Domain Protein | EgrG_000255800 | 267 | 4 | 4 |
| Tetraspanin | EgrG_000354700 | 27 | 3 | 3 |
| Expressed Conserved Protein | EgrG_001085900 | 23 | 3 | 6 |
| Syndecan Binding Protein Syntenin | EgrG_000453900 | 31 | 3 | 4 |
| Major Egg Antigen P40 | EgrG_000212700 | 36 | 3 | 3 |
| Expressed Protein | EgrG_000513800 | 33 | 3 | 3 |
| Expressed Conserved Protein | EgrG_000756700 | 57 | 3 | 3 |
| Ribosomal Protein S3 | EgrG_001167300 | 26 | 3 | 3 |
| Dynein Light Chain | EgrG_000182400 | 19 | 3 | 4 |
| Expressed Conserved Protein | EgrG_000859100 | 29 | 3 | 4 |
| Thioredoxin Glutathione Reductase | EgrG_000222300 | 58 | 3 | 3 |
| Citrate Synthase | EgrG_001028500 | 51 | 3 | 3 |
| Gelsolin | EgrG_000882300 | 42 | 3 | 3 |
| Dynein Light Chain | EgrG_000990900 | 11 | 3 | 4 |
| Malate Dehydrogenase | EgrG_000417100 | 37 | 3 | 3 |
| Dynein Light Chain | EgrG_000941000 | 22 | 3 | 3 |
| Enolase | EGR_07685 | 115 | 3 | 3 |
| Diagnostic Antigen Gp50 (Fragment) | EgrG_000564000 | 22 | 2 | 3 |
| Tctex1 Domain-Containing Protein | EGR_07068 | 50 | 2 | 2 |
| Uncharacterized Protein | EGR_03661 | 8 | 2 | 2 |
| Tapeworm Specific Antigen B | EgrG_000381400 | 10 | 2 | 2 |
| Dynein Light Chain Type 1 2 | EgrG_000991300 | 10 | 2 | 2 |
| Antigen B 1/1 (Fragment) | EgrG_000381100 | 7 | 2 | 3 |
| Vesicle Associated Membrane Protein | EgrG_000494400 | 31 | 2 | 2 |
| Myoferlin | EgrG_000825200 | 230 | 2 | 2 |
| Glyceraldehyde-3-Phosphate Dehydrogenase | EgrG_000254600 | 38 | 2 | 2 |
| Casein Kinase I Alpha | EgrG_000815700 | 42 | 2 | 2 |
| Tetraspanin | EgrG_000834300 | 25 | 2 | 2 |
| Tyrosine-Protein Kinase | EgrG_000186700 | 105 | 2 | 2 |
| Dynein Light Chain 1, Cytoplasmic | EGR_04637 | 12 | 2 | 3 |
| Annexin | EgrG_000243700 | 34 | 2 | 2 |
| Lysyl Oxidase | EGR_08654 | 58 | 2 | 2 |
| Neutral Amino Acid Transporter A | EgrG_001168200 | 33 | 2 | 2 |
| Expressed Protein | EgrG_000724500 | 9 | 2 | 2 |
| Uncharacterized Protein | EGR_08255 | 38 | 2 | 3 |
| Calpain | EgrG_000719700 | 86 | 2 | 2 |
| Uncharacterized Protein | EGR_00893 | 48 | 2 | 2 |
| Serine/Threonine-Protein Phosphatase | EgrG_000779500 | 43 | 2 | 2 |
| Expressed Conserved Protein | EgrG_000682000 | 24 | 2 | 2 |
| Ubiquitin Family Member Ubq 1 | EgrG_001086700 | 39 | 2 | 2 |
| 14-3-3 Protein | EgrG_001192500 | 28 | 2 | 2 |
| Dnaj Subfamily B | EgrG_000429950 | 38 | 2 | 2 |
| Rab | EgrG_000349500 | 24 | 2 | 2 |
| Annexin | EgrG_000041300 | 37 | 2 | 2 |
| Alpha Glucosidase | EgrG_000494800 | 68 | 2 | 2 |
| Tegumental Antigen | EgrG_001001400 | 24 | 2 | 2 |
| Laminin | EgrG_001132400 | 395 | 2 | 2 |
| Expressed Protein | EgrG_000860500 | 97 | 2 | 2 |
