# Supplementary material for: Identification of Different Extracellular Vesicles in the Hydatid Fluid of Echinococcus granulosus and Immunomodulatory Effects of 110 K EVs on Sheep PBMCs
Source: Front Immunol. 2021 Feb 23;12:602717. doi: 10.3389/fimmu.2021.602717 (PMC7940240; doi:10.3389/fimmu.2021.602717)
Supplement: Supplementary file 3 [file Table_3.DOCX]

Table S3 Protein cargoes of *E. granulosus* HF 110 K EVs

| **Protein** | **Protein ID** | **Size (kDa)** | **Unique Peptide** | **Unique Spectra** |
| --- | --- | --- | --- | --- |
| Myoferlin | EgrG_000825200 | 230 | 86 | 3 |
| Basement Membrane Specific Heparan Sulfate | EgrG_000575900 | 859 | 81 | 1 |
| Lipid Transport Protein N Terminal | EgrG_000684200 | 344 | 53 | 1 |
| Otoferlin | EgrG_000436000 | 244 | 42 | 1 |
| Major Vault Protein | EgrG_000142500 | 97 | 31 | 2 |
| Calpain | EgrG_000719700 | 85 | 30 | 12 |
| Programmed Cell Death 6 Interacting Protein | EgrG_000997550 | 89 | 29 | 1 |
| Phosphoenolpyruvate Carboxykinase | EgrG_000292700 | 71 | 29 | 9 |
| Atp Binding Cassette Subfamily B Mdr:Tap | EgrG_000901000 | 132 | 29 | 1 |
| Multidrug Resistance Associated Protein 1 | EgrG_000592100 | 220 | 29 | 2 |
| Estrogen Regulated Protein Ep45 | EgrG_000824100 | 50 | 28 | 1 |
| Anoctamin-1 | EgrG_000175600 | 117 | 27 | 1 |
| Nedd4 E3 Ubiquitin Protein Ligase Wwp1 | EgrG_000163200 | 95 | 27 | 1 |
| Calpain-A | EgrG_000911200 | 92 | 27 | 2 |
| Atp Binding Cassette Subfamily B Mdr:Tap | EgrG_000901300 | 139 | 27 | 5 |
| Phospholipid Transporting Atpase Iib | EgrG_000172600 | 120 | 26 | 1 |
| Atrial Natriuretic Peptide Receptor 1 | EgrG_000928050 | 101 | 25 | 46 |
| Annexin | EgrG_000244000 | 37 | 24 | 2 |
| Glycoprotein Antigen 5 | EgrG_000184900 | 55 | 22 | 1 |
| Filamin | EgrG_000859700 | 249 | 21 | 2 |
| Phospholipase D | EgrG_000249000 | 165 | 21 | 1 |
| Hypothetical Protein | EgrG_001115400 | 95 | 21 | 8 |
| Annexin | EgrG_000041200 | 39 | 20 | 1 |
| Na:K Atpase Alpha Subunit | EgrG_000342600 | 113 | 20 | 1 |
| Lysosomal Alpha Glucosidase | EgrG_000143500 | 101 | 19 | 2 |
| Sodium Driven Chloride Bicarbonate Exchanger | EgrG_000319700 | 121 | 18 | 2 |
| Telomerase Protein Component 1 | EgrG_001036600 | 308 | 17 | 2 |
| Annexin | EgrG_000193700 | 39 | 16 | 5 |
| Annexin A13 | EgrG_000041300 | 37 | 16 | 9 |
| Tissue Type Plasminogen Activator | EgrG_000096600 | 239 | 16 | 2 |
| Basement Membrane Specific Heparan Sulfate | EgrG_000701800 | 96 | 16 | 4 |
| H17G Protein Tegumental Antigen | EgrG_000485800 | 71 | 16 | 1 |
| Dynein Heavy Chain | EgrG_000832000 | 535 | 16 | 6 |
| Prominin Protein | EgrG_000057700 | 107 | 15 | 15 |
| T Cell Immunomodulatory Protein | EgrG_000440000 | 64 | 15 | 7 |
| Neurogenic Locus Notch Protein | EgrG_000343000 | 339 | 15 | 1 |
| Hypothetical Protein | EgrG_001110400 | 118 | 15 | 1 |
| Leucine Rich Repeat Containing Protein 16C, Partial | EgrG_000685100 | 98 | 15 | 1 |
| Laminin | EgrG_001132400 | 395 | 15 | 2 |
| Annexin | EgrG_000243600 | 35 | 15 | 1 |
| Bro1 Domain Containing Protein Brox | EgrG_000524700 | 49 | 14 | 1 |
| Dipeptidyl Peptidase 3 | EgrG_001028100 | 83 | 14 | 1 |
| Phospholipid Transporting Atpase If | EgrG_000079100 | 150 | 14 | 1 |
| Syntaxin Binding Protein 1 | EgrG_001188600 | 77 | 14 | 2 |
| Retinal Guanylyl Cyclase 2 | EgrG_000504700 | 130 | 14 | 3 |
| Expressed Conserved Protein | EgrG_001018600 | 62 | 14 | 1 |
| Sodium And Chloride Dependent Glycine | EgrG_000220200 | 87 | 14 | 1 |
| Ubiquitin Carboxyl Terminal Hydrolase 4 | EgrG_000915300 | 169 | 14 | 1 |
| Acetylcholinesterase | EgrG_001075400 | 71 | 13 | 13 |
| Thioredoxin Glutathione Reductase | EgrG_000222300 | 58 | 13 | 4 |
| Ribosomal Protein S1 Rna Binding Domain | EgrG_000975800 | 98 | 13 | 13 |
| Glutamate Receptor Ionotropic Kainate | EgrG_001066000 | 90 | 13 | 3 |
| Tyrosine Protein Phosphatase Non Receptor Type | EgrG_000945550 | 192 | 13 | 4 |
| Enolase | EgrG_000514200 | 47 | 13 | 1 |
| Heat Shock Cognate Protein | EgrG_001085400 | 71 | 12 | 1 |
| Ubiquitin Family Member Ubq 1 | EgrG_001086700 | 39 | 12 | 1 |
| Expressed Conserved Protein | EgrG_001061900 | 74 | 12 | 16 |
| Syntenin-1 | EgrG_000453900 | 31 | 12 | 1 |
| Alpha Actinin Sarcomeric | EgrG_000856100 | 108 | 12 | 4 |
| Neurexin 4 | EgrG_000215800 | 134 | 12 | 4 |
| Ap 2 Complex Subunit Alpha 2 | EgrG_001050200 | 107 | 12 | 2 |
| Serine Protease Inhibitor | EgrG_001193200 | 39 | 12 | 5 |
| Severin | EgrG_000882300 | 42 | 12 | 3 |
| Solute Carrier Family 12 | EgrG_000080600 | 135 | 12 | 1 |
| Von Willebrand Factor A Domain Containing Protein | EgrG_001168700 | 88 | 11 | 1 |
| Actin Modulator Protein | EgrG_000882500 | 46 | 11 | 6 |
| Ornithine Aminotransferase | EgrG_001032200 | 46 | 11 | 8 |
| Puromycin Sensitive Aminopeptidase | EgrG_001105200 | 103 | 11 | 1 |
| Transketolase | EgrG_000103100 | 68 | 11 | 3 |
| Hypothetical Protein | EgrG_000264700 | 100 | 11 | 17 |
| Huntingtin Interacting Protein 1 | EgrG_000728100 | 101 | 11 | 1 |
| Beta Mannosidase | EgrG_000789900 | 108 | 11 | 1 |
| Dehydrogenase: Reductase Sdr Family | EgrG_000410100 | 45 | 11 | 1 |
| Polycystic Kidney Disease Protein | EgrG_000622700 | 111 | 11 | 9 |
| Calpain-7 | EgrG_001000650 | 90 | 11 | 59 |
| Collagen Alpha 1V Chain | EgrG_000144300 | 177 | 10 | 1 |
| Galectin Carbohydrate Recognition Domain | EgrG_000226500 | 139 | 10 | 5 |
| Epidermal Growth Factor Receptor Kinase | EgrG_000099700 | 61 | 10 | 1 |
| Tetraspanin | EgrG_000355700 | 30 | 10 | 3 |
| Major Egg Antigen | EgrG_000236500 | 36 | 10 | 1 |
| Plasma Membrane Calcium Transporting Atpase 2 | EgrG_001191500 | 130 | 10 | 2 |
| Atp Binding Cassette Sub Family A | EgrG_000519900 | 211 | 10 | 2 |
| Hypothetical Protein | EgrG_000242800 | 95 | 10 | 2 |
| Peroxidasin | EgrG_000733600 | 149 | 10 | 1 |
| V-Type Proton Atpase Subunit A Isoform | EgrG_000227800 | 101 | 10 | 3 |
| Collagen Alpha 1(Iv) Chain | EgrG_000144350 | 172 | 10 | 20 |
| Prosaposin A Preproprotein | EgrG_000733100 | 114 | 10 | 2 |
| Annexin | EgrG_000243700 | 34 | 10 | 8 |
| Phosphoglycerate Kinase 1 | EgrG_001043100 | 47 | 10 | 6 |
| Cathepsin D Lysosomal Aspartyl Protease | EgrG_000970500 | 47 | 10 | 4 |
| Phospholipid Scramblase 3 | EgrG_000624400 | 34 | 10 | 1 |
| Cyclin Y | EgrG_001021100 | 47 | 10 | 3 |
| Rab Gdp Dissociation Inhibitor Alpha | EgrG_000767200 | 50 | 10 | 3 |
| Glucose 6 Phosphate Isomerase | EgrG_000626300 | 62 | 9 | 4 |
| Tnf Receptor Associated Factor 3 | EgrG_000548900 | 54 | 9 | 16 |
| Vacuolar Protein Sorting-Associated Protein 4A | EgrG_000486200 | 49 | 9 | 5 |
| Tubulin Beta-3 Chain | EgrG_000202500 | 50 | 9 | 1 |
| 2'3' Cyclic Nucleotide | EgrG_001200600 | 54 | 9 | 4 |
| Ap 1 Complex Subunit Beta 1 | EgrG_000240100 | 107 | 9 | 13 |
| Polyubiquitin | EgrG_000516500 | 15 | 9 | 6 |
| Hypothetical Protein | EgrG_000303000 | 111 | 9 | 23 |
| Transient Receptor Potential Gamma Protein | EgrG_000908800 | 113 | 9 | 1 |
| Vesicular Fusion Protein Nsf | EgrG_000338600 | 81 | 9 | 4 |
| Phospholipid Translocating Atpase | EgrG_000531500 | 145 | 9 | 5 |
| Fructose-Bisphosphate Aldolase | EgrG_000905600 | 40 | 9 | 37 |
| Expressed Conserved Protein | EgrG_000531300 | 51 | 8 | 1 |
| Solute Carrier Family 5 | EgrG_000714050 | 75 | 8 | 8 |
| Dnaj Subfamily A | EgrG_000101800 | 44 | 8 | 1 |
| Camp-Dependent Protein Kinase Type Ii Regulatory Subunit | EgrG_000775600 | 42 | 8 | 3 |
| Synaptotagmin 2 | EgrG_001009500 | 46 | 8 | 1 |
| Elongation Factor 1 Alpha | EgrG_000982200 | 50 | 8 | 2 |
| Tsp1 | EgrG_000355800 | 29 | 8 | 18 |
| Expressed Conserved Protein | EgrG_000756700 | 57 | 8 | 5 |
| Calcium Binding Protein P22 | EgrG_000447500 | 21 | 8 | 1 |
| Egf Domain Protein | EgrG_000255800 | 267 | 8 | 1 |
| Cytosolic Malate Dehydrogenase | EgrG_000417100 | 37 | 8 | 3 |
| Polyu Specific Endoribonuclease | EgrG_001132700 | 29 | 8 | 6 |
| Expressed Protein | EgrG_000305900 | 50 | 8 | 5 |
| Retinal Guanylyl Cyclase 2 | EgrG_000554500 | 100 | 8 | 78 |
| Transitional Endoplasmic Reticulum Atpase | EgrG_000471600 | 88 | 8 | 24 |
| Alpha Glucosidase | EgrG_000494800 | 68 | 8 | 1 |
| Seryl-Trna Synthetase, Cytoplasmic | EgrG_001197300 | 63 | 8 | 1 |
| Aldehyde Dehydrogenase Family 1 Member A3 | EgrG_000389100 | 52 | 7 | 19 |
| C Type Lectin Domian Containing Protein | EgrG_000206400 | 59 | 7 | 2 |
| Zinc Finger C2H2 Type | EgrG_000639300 | 53 | 7 | 6 |
| Laminin Subunit Beta | EgrG_000880000 | 194 | 7 | 4 |
| Neutral Alpha Glucosidase Ab | EgrG_000716600 | 110 | 7 | 1 |
| Cadherin Egf Lag Seven Pass G Type Receptor 1 | EgrG_000665500 | 255 | 7 | 5 |
| Maspardin | EgrG_000725100 | 32 | 7 | 9 |
| Cd36 Class B Scavenger Receptor | EgrG_000637200 | 58 | 7 | 4 |
| Mechanosensory Protein 2 | EgrG_000205700 | 53 | 7 | 15 |
| Long Chain Fatty Acid Coenzyme A Ligase 1 | EgrG_000708900 | 77 | 7 | 1 |
| Guanine Nucleotide Binding Protein Subunit | EgrG_000200300 | 37 | 7 | 4 |
| Endophilin B1 | EgrG_000550800 | 29 | 7 | 8 |
| Glycogen Synthase | EgrG_001029800 | 95 | 7 | 34 |
| Heat Shock Protein Hsp 90-Alpha | EgrG_000008700 | 74 | 7 | 1 |
| Phospholipid Scramblase 2 | EgrG_000518150 | 30 | 7 | 3 |
| Lysosomal Alpha Mannosidase | EgrG_000704400 | 118 | 7 | 4 |
| Asparaginyl Trna Synthetase, Cytoplasmic | EgrG_002026800 | 117 | 7 | 4 |
| Gamma Glutamyltransferase 1 | EgrG_000761500 | 84 | 7 | 7 |
| P2X Purinoceptor 4 | EgrG_000977600 | 50 | 7 | 2 |
| Cgmp Dependent Protein Kinase | EgrG_000158500 | 111 | 7 | 1 |
| Dihydropyrimidinase | EgrG_000953400 | 64 | 7 | 2 |
| Neuroglian | EgrG_000240400 | 121 | 7 | 2 |
| Tegumental Protein | EgrG_000059800 | 21 | 7 | 2 |
| Lysosomal Acid Lipase:Cholesteryl Ester | EgrG_000357600 | 45 | 7 | 1 |
| Hypothetical Protein | EgrG_000302100 | 49 | 7 | 1 |
| Glycogenin 1 | EgrG_000801500 | 105 | 7 | 2 |
| Ribosomal Protein S1 Rna Binding Domain | EgrG_000967800 | 107 | 7 | 2 |
| Annexin | EgrG_000925300 | 40 | 6 | 19 |
| Intestinal Type Alkaline Phosphatase 1 | EgrG_000393300 | 60 | 6 | 1 |
| Otopetrin | EgrG_000449800 | 115 | 6 | 2 |
| Ethe1 Protein | EgrG_001090400 | 30 | 6 | 4 |
| Heat Shock Protein 71 Kda Protein | EgrG_001085100 | 73 | 6 | 1 |
| Hypothetical Protein | EgrG_000849600 | 31 | 6 | 2 |
| Expressed Protein | EgrG_000260400 | 35 | 6 | 2 |
| Expressed Protein | EgrG_000305200 | 59 | 6 | 1 |
| Prolyl Endopeptidase | EgrG_000668800 | 81 | 6 | 23 |
| Myotubularin Protein 13 | EgrG_000857000 | 286 | 6 | 23 |
| Utp--Glucose-1-Phosphate Uridylyltransferase | EgrG_000843500 | 57 | 6 | 1 |
| Hypothetical Protein | EgrG_000658900 | 118 | 6 | 2 |
| Threonyl-Trna Synthetase, Cytoplasmic | EgrG_000375800 | 83 | 6 | 17 |
| Glycogen Phosphorylase | EgrG_000501600 | 98 | 6 | 8 |
| Glyceraldehyde 3 Phosphate Dehydrogenase | EgrG_000254600 | 38 | 6 | 129 |
| Putative 14-3-3 Protein | EgrG_001192500 | 28 | 6 | 1 |
| Expressed Conserved Protein | EgrG_000858600 | 52 | 6 | 1 |
| Multivesicular Body Subunit 12B | EgrG_000409800 | 25 | 6 | 1 |
| Vacuolar Protein Sorting-Associated Protein | EgrG_000675600 | 28 | 6 | 9 |
| Vesicle Associated Membrane Protein | EgrG_000494400 | 31 | 6 | 5 |
| Casein Kinase I Alpha | EgrG_000815700 | 42 | 6 | 5 |
| Collagen Alpha 2I Chain | EgrG_000823800 | 131 | 6 | 1 |
| Phosphatidate Phosphatase | EgrG_000635000 | 29 | 6 | 2 |
| Transient Receptor Potential Cation Channel | EgrG_000175000 | 272 | 6 | 1 |
| Proto Oncogene Tyrosine Protein Kinase Lck | EgrG_000670400 | 60 | 6 | 1 |
| Ras Protein Rab 7A | EgrG_000244200 | 23 | 6 | 1 |
| Beta Ureidopropionase | EgrG_000482700 | 42 | 6 | 2 |
| V-Type Proton Atpase Subunit D | EgrG_000617100 | 41 | 6 | 132 |
| Ras-Related Protein Rab-11A | EgrG_000349500 | 24 | 6 | 7 |
| Thioredoxin-Dependent Peroxide Reductase | EgrG_000791700 | 21 | 6 | 3 |
| Synaptotagmin | EgrG_000470900 | 59 | 6 | 3 |
| Cd109 Antigen | EgrG_000610100 | 182 | 6 | 7 |
| Lanc Protein | EgrG_001073250 | 47 | 6 | 2 |
| Fibrillar Collagen Chain Fap1 Alpha | EgrG_001060700 | 115 | 6 | 2 |
| Annexin | EgrG_000330300 | 48 | 6 | 1 |
| Excitatory Amino Acid Transporter 3 | EgrG_001182500 | 53 | 6 | 1 |
| Aspartate Aminotransferase Mitochondrial | EgrG_001134100 | 47 | 6 | 2 |
| 14-3-3 Protein Zeta | EgrG_000231300 | 29 | 6 | 2 |
| Contactin | EgrG_000694600 | 136 | 6 | 2 |
| Ferritin Heavy Chain | EgrG_000382200 | 20 | 6 | 2 |
| Endophilin A3 | EgrG_001064500 | 38 | 6 | 23 |
| Protein Disulfide Isomerase A3 | EgrG_001022300 | 81 | 5 | 6 |
| Protein Kinase C And Casein Kinase Substrate In | EgrG_001090800 | 84 | 5 | 2 |
| Tubulin Alpha Chain | EgrG_000886400 | 57 | 5 | 1 |
| Palmitoyltransferase Zdhhc20 | EgrG_001200400 | 40 | 5 | 3 |
| Ubx Domain-Containing Protein 6 | EgrG_000221200 | 53 | 5 | 1 |
| Hemicentin 1 | EgrG_000422350 | 476 | 5 | 114 |
| Protein Of Unknown Function Duf292 Eukaryotic | EgrG_000661100 | 36 | 5 | 1 |
| Trehalose 6 Phosphate Hydrolase | EgrG_000753100 | 78 | 5 | 7 |
| Iron:Zinc Purple Acid Phosphatase Protein | EgrG_001169400 | 54 | 5 | 7 |
| Clathrin Heavy Chain | EgrG_000122200 | 191 | 5 | 1 |
| Long Chain Fatty Acid Coenzyme A Ligase 1 | EgrG_000457800 | 71 | 5 | 8 |
| Dendritic Cell Derived Ubiquitin | EgrG_000759900 | 40 | 5 | 8 |
| Tetraspanin | EgrG_000354700 | 27 | 5 | 3 |
| Cyclosporin A-Binding Protein | EgrG_000920600 | 17 | 5 | 2 |
| Small Heat Shock Protein P36 | EgrG_000212700 | 36 | 5 | 8 |
| Sodium Coupled Monocarboxylate Transporter 1 | EgrG_000636200 | 69 | 5 | 3 |
| Cathepsin L | EgrG_000967900 | 41 | 5 | 19 |
| N Ethylmaleimide Sensitive Factor Attachment | EgrG_000906800 | 30 | 5 | 2 |
| Ankyrin Repeat And Death Domain Containing Protein | EgrG_000363900 | 95 | 5 | 7 |
| Udp Glucose 4 Epimerase | EgrG_000984800 | 39 | 5 | 5 |
| Stip1Y And U Box-Containing Protein | EgrG_000604500 | 38 | 5 | 1 |
| Leukocyte Surface Antigen Cd53 | EgrG_001077100 | 24 | 5 | 16 |
| E3 Ubiquitin Protein Ligase Herc4 | EgrG_000424000 | 138 | 5 | 1 |
| Aminopeptidase N | EgrG_001063900 | 115 | 5 | 15 |
| Enteropeptidase | EgrG_000085400 | 51 | 5 | 4 |
| Serine:Threonine Protein Kinase N2 | EgrG_000109100 | 122 | 5 | 8 |
| Heat Shock Protein 105, Partial | EgrG_000917000 | 71 | 5 | 2 |
| Ral-Like Protein | EgrG_000807900 | 23 | 5 | 8 |
| Actin Protein 3B | EgrG_000292600 | 51 | 5 | 8 |
| Tyrosine Protein Kinase Src42A | EgrG_000598200 | 72 | 5 | 4 |
| Diagnostic Antigen Gp50 | EgrG_000566700 | 27 | 5 | 1 |
| Synaptic Vesicle Membrane Protein Vat-1-Like Protein | EgrG_000935200 | 47 | 5 | 1 |
| Family S60 Non Peptidase Ue S60 Family | EgrG_000569300 | 85 | 5 | 1 |
| Multidrug Resistance Protein 1 | EgrG_000160100 | 60 | 5 | 4 |
| Aldehyde Dehydrogenase Family 3 Member B1 | EgrG_000583800 | 38 | 5 | 3 |
| Long Chain Fatty Acid Coenzyme A Ligase 4 | EgrG_000376500 | 89 | 5 | 7 |
| Cd36 Class B Scavenger Receptor | EgrG_000657850 | 60 | 5 | 191 |
| Expressed Conserved Protein | EgrG_001158800 | 37 | 5 | 1 |
| Actin-1 | EgrG_000406900 | 42 | 5 | 10 |
| Cd9 Antigen | EgrG_000833400 | 28 | 5 | 4 |
| Casein Kinase Ii Subunit Alpha | EgrG_000457900 | 53 | 5 | 5 |
| Ras-Related Protein Rab-2A | EgrG_000430800 | 23 | 5 | 105 |
| Ubiquitin Modifier Activating Enzyme 1 | EgrG_000711500 | 121 | 5 | 6 |
| Fimbrin | EgrG_000786800 | 69 | 5 | 2 |
| Endonuclease:Exonuclease:Phosphatase Family | EgrG_000661600 | 52 | 5 | 4 |
| Maguk P55 Subfamily | EgrG_000494200 | 95 | 5 | 2 |
| Vacuolar Protein Sorting Associated Protein 37B | EgrG_000745800 | 23 | 4 | 10 |
| Ubiquitin Carboxyl Terminal Hydrolase 4 | EgrG_000065800 | 92 | 4 | 1 |
| Sulfhydryl Oxidase 1 | EgrG_000245600 | 65 | 4 | 6 |
| Cathepsin B-Like Peptidase | EgrG_000790200 | 39 | 4 | 74 |
| Gamma Soluble Nsf Attachment Protein | EgrG_000924400 | 34 | 4 | 1 |
| Tubulin Alpha-1C Chain | EgrG_000413200 | 50 | 4 | 3 |
| Aspartyl-Trna Synthetase, Cytoplasmic | EgrG_000777100 | 58 | 4 | 9 |
| Protein Disulfide Isomerase | EgrG_000094600 | 59 | 4 | 80 |
| Serine:Threonine Protein Phosphatase Pp1 Beta | EgrG_000522800 | 37 | 4 | 2 |
| Eh Domain-Containing Protein | EgrG_000851900 | 64 | 4 | 7 |
| Apolipoprotein A I Binding Protein | EgrG_000736050 | 41 | 4 | 4 |
| Calcium:Calmodulin Dependent 3'5' Cyclic | EgrG_000363800 | 78 | 4 | 77 |
| T Complex Protein 1 Subunit Gamma | EgrG_000872100 | 59 | 4 | 10 |
| Elongation Factor 1-Gamma | EgrG_000113800 | 48 | 4 | 4 |
| T-Complex Protein 1 Subunit Zeta | EgrG_000143600 | 60 | 4 | 2 |
| Charged Multivesicular Body Protein | EgrG_000468500 | 23 | 4 | 3 |
| Dnaj Subfamily B | EgrG_000614200 | 40 | 4 | 3 |
| Cc2D1B Protein | EgrG_000693400 | 92 | 4 | 11 |
| Fructose 16 Bisphosphatase 1 | EgrG_000261600 | 40 | 4 | 1 |
| Tetraspanin | EgrG_000355400 | 30 | 4 | 9 |
| Myosin Xv | EgrG_001038000 | 241 | 4 | 1 |
| Solute Carrier Family 2 Facilitated Glucose | EgrG_000972600 | 53 | 4 | 4 |
| Ras Protein Rab 10 | EgrG_001176700 | 27 | 4 | 2 |
| Guanine Nucleotide Binding Protein G Protein | EgrG_001157400 | 45 | 4 | 16 |
| Glycogen Phosphorylase | EgrG_000501500 | 98 | 4 | 1 |
| F-Actin-Capping Protein Subunit Beta Isoform | EgrG_000772300 | 31 | 4 | 1 |
| Inorganic Pyrophosphatase | EgrG_000487400 | 34 | 4 | 6 |
| Zinc Finger Ring Type | EgrG_000844600 | 79 | 4 | 3 |
| Pdz Domain Containing Protein | EgrG_000105600 | 35 | 4 | 2 |
| Endoglycoceramidase | EgrG_000059500 | 58 | 4 | 1 |
| Gynecophoral Canal Protein | EgrG_000712600 | 97 | 4 | 1 |
| Dynamin | EgrG_000105900 | 99 | 4 | 1 |
| Alkaline Phosphatase | EgrG_000393400 | 61 | 4 | 1 |
| Hypothetical Protein | EgrG_000340400 | 45 | 4 | 1 |
| Mfs Transporter Ceroid Lipofuscinosis Neuronal | EgrG_000843800 | 54 | 4 | 5 |
| Tsp | EgrG_001070300 | 23 | 4 | 23 |
| Major Facilitator Superfamily General Substrate Transporter | EgrG_001051700 | 144 | 4 | 16 |
| Long Chain Fatty Acid Coenzyme A Ligase 5 | EgrG_000078800 | 86 | 4 | 2 |
| Nardilysin | EgrG_000935900 | 131 | 4 | 2 |
| Calpain 5 | EgrG_000205500 | 77 | 4 | 3 |
| Growth Factor Receptor Bound Protein 7 | EgrG_000263700 | 56 | 4 | 15 |
| Abnormal Embroygenesis Family Member Emb 9 | EgrG_000144400 | 168 | 4 | 6 |
| Ribose 5 Phosphate Isomerase | EgrG_001185300 | 29 | 4 | 1 |
| Syntaxin Binding Protein 1 | EgrG_000380500 | 70 | 4 | 1 |
| Potassium Voltage Gated Channel Subfamily D | EgrG_000570000 | 84 | 4 | 2 |
| Oxalate:Formate Antiporter | EgrG_000661800 | 53 | 4 | 4 |
| Ras Protein Rab 27A | EgrG_000347300 | 35 | 4 | 2 |
| Hypothetical Protein Egr_04177 | EgrG_000127000 | 15 | 4 | 1 |
| Coronin-1B | EgrG_001056750 | 56 | 4 | 3 |
| Spectrin Alpha Chain | EgrG_000151600 | 282 | 4 | 3 |
| Choline Transporter Protein 2 | EgrG_000203700 | 82 | 4 | 9 |
| Sodium:Dicarboxylate Symporter | EgrG_000469500 | 53 | 4 | 4 |
| Synaptic Vesicle 2 Protein | EgrG_000580600 | 67 | 4 | 15 |
| Fatty Aldehyde Dehydrogenase | EgrG_000608100 | 52 | 4 | 21 |
| Tumor Susceptibility Gene 101 Protein | EgrG_000664100 | 63 | 4 | 1 |
| ATP Binding Cassette Sub Family G | EgrG_000976200 | 106 | 4 | 1 |
| Adp Ribosylation Factor 6 | EgrG_001006100 | 20 | 4 | 2 |
| Sh3 And Multiple Ankyrin Repeat Domains Protein | EgrG_001127100 | 116 | 4 | 1 |
| Ww Domain Binding Protein 2 | EgrG_000689500 | 35 | 4 | 2 |
| Phosphoglycerate Mutase | EgrG_000799500 | 34 | 4 | 2 |
| Hypothetical Protein Egrg_001089200 | EgrG_001089200 | 40 | 4 | 3 |
| Expressed Conserved Protein | EgrG_000701500 | 342 | 4 | 7 |
| Procollagen Galactosyltransferase | EgrG_000476300 | 71 | 4 | 6 |
| Phosphatidylinositol 5 Phosphate 4 Kinase Type 2 | EgrG_000394700 | 76 | 4 | 5 |
| Protein Dj-1 | EgrG_000135900 | 19 | 4 | 14 |
| Chaperonin Containing Tcp1 Subunit 2 Beta | EgrG_000702700 | 67 | 4 | 2 |
| Oxalate:Formate Antiporter | EgrG_000662500 | 51 | 4 | 6 |
| Putative Citrate Synthase | EgrG_001028500 | 51 | 4 | 1 |
| Proteasome Subunit Alpha Type-7-Like Protein | EgrG_000682700 | 30 | 4 | 1 |
| Annexin | EgrG_000237700 | 36 | 4 | 2 |
| Spry Domain Containing Protein | EgrG_000446700 | 137 | 4 | 13 |
| Myosin Heavy Chain Non Muscle | EgrG_000479300 | 227 | 4 | 1 |
| Syntaxin | EgrG_000746800 | 33 | 4 | 7 |
| Nervana 2 | EgrG_000330900 | 34 | 4 | 4 |
| Monocarboxylate Transporter | EgrG_000606100 | 53 | 4 | 32 |
| Tubulin Beta 2C Chain | EgrG_000672200 | 50 | 4 | 1 |
| Phospholipid Hydroperoxide Glutathione | EgrG_000374800 | 28 | 4 | 1 |
| Fras1 Related Extracellular Matrix Protein | EgrG_000086300 | 272 | 4 | 1 |
| Glutamate Receptor Ionotropic Kainate 3 | EgrG_000849400 | 91 | 4 | 1 |
| Thioredoxin Fold | EgrG_000666500 | 14 | 4 | 3 |
| Putative Growth Regulator 14-3-3 | EgrG_000364000 | 28 | 4 | 2 |
| Endophilin B2 | EgrG_000060900 | 29 | 4 | 4 |
| Exocyst Complex Component 1 | EgrG_001048900 | 95 | 4 | 16 |
| Potassium Large Conductance Calcium Activated | EgrG_001146300 | 144 | 4 | 11 |
| Prostaglandin H2 D Isomerase | EgrG_000459050 | 24 | 4 | 1 |
| Ectonucleoside Triphosphate Diphosphohydrolase | EgrG_000828300 | 37 | 4 | 1 |
| Transaldolase | EgrG_000092800 | 37 | 4 | 1 |
| Actin Cytoplasmic Type 5 | EgrG_000190400 | 40 | 4 | 9 |
| Solute Carrier Family 2 Facilitated Glucose | EgrG_000853500 | 55 | 3 | 1 |
| Tetraspanin Family Protein 16 Invertebrate | EgrG_000834300 | 25 | 3 | 5 |
| Nadh Ubiquinone Oxidoreductase Subunit 10 | EgrG_000096100 | 151 | 3 | 2 |
| Phosphatidylinositol Transfer Protein Alpha Isoform | EgrG_000301100 | 30 | 3 | 10 |
| Transforming Growth Factor-Beta-Induced Protein Ig-H3 | EgrG_000824400 | 73 | 3 | 1 |
| Transforming Protein Rhoa | EgrG_000246600 | 27 | 3 | 1 |
| Heat Shock Protein Family Member Hsp 3 | EgrG_000249600 | 72 | 3 | 1 |
| Polycystic Kidney Disease Protein 2 | EgrG_001123300 | 91 | 3 | 3 |
| Zinc Carboxypeptidase Family Protein | EgrG_000421900 | 58 | 3 | 14 |
| Dynein Light Chain 1, Cytoplasmic | EgrG_000991200 | 12 | 3 | 1 |
| Ankyrin Repeat Domain-Containing Protein 13C-B | EgrG_000673600 | 47 | 3 | 1 |
| Ras Protein Rap 1B | EgrG_000207300 | 25 | 3 | 1 |
| Gtp Binding Protein Cg1354 | EgrG_000677800 | 46 | 3 | 2 |
| Phosphatidylinositol Binding Clathrin Assembly | EgrG_001107500 | 72 | 3 | 1 |
| Estrogen Regulated Protein Ep45 | EgrG_000824000 | 45 | 3 | 1 |
| Carbonic Anhydrase Ii | EgrG_000535400 | 35 | 3 | 7 |
| Expressed Conserved Protein | EgrG_001159100 | 28 | 3 | 1 |
| Hypothetical Protein | EgrG_000740700 | 29 | 3 | 6 |
| Aldo Keto Reductase Family 1 Member B4 | EgrG_000156400 | 42 | 3 | 1 |
| Adp-Ribosylation Factor 4 | EgrG_001177600 | 20 | 3 | 2 |
| Ras Protein Rabf2B | EgrG_001004250 | 20 | 3 | 14 |
| Lysosomal Pro X Carboxypeptidase | EgrG_000456150 | 53 | 3 | 5 |
| Solute Carrier Family 13 | EgrG_000597400 | 54 | 3 | 5 |
| Tsp6 | EgrG_001021300 | 24 | 3 | 1 |
| Non Lysosomal Glucosylceramidase | EgrG_000876700 | 110 | 3 | 5 |
| Rho Gdp Dissociation Inhibitor | EgrG_001152900 | 24 | 3 | 8 |
| Ste20 Serine:Threonine Protein Kinase | EgrG_001193000 | 117 | 3 | 1 |
| Triosephosphate Isomerase | EgrG_000416400 | 27 | 3 | 1 |
| Proteasome Subunit Alpha Type-3 | EgrG_000196100 | 28 | 3 | 7 |
| Synaptobrevin Ykt6 | EgrG_000826400 | 36 | 3 | 3 |
| Epidermal Growth Factor Receptor Kinase | EgrG_000203000 | 96 | 3 | 1 |
| Synaptosomal Associated Protein | EgrG_000412200 | 27 | 3 | 1 |
| Beta 13 N Galactosyltransferase | EgrG_000545700 | 43 | 3 | 1 |
| Major Egg Antigen P40 | EgrG_000236400 | 52 | 3 | 8 |
| Phox | EgrG_001037300 | 58 | 3 | 21 |
| Chromatin Regulatory Protein Sir2 | EgrG_001065100 | 44 | 3 | 6 |
| Glycogenin 1 | EgrG_000194000 | 47 | 3 | 12 |
| Calpain-B | EgrG_000319100 | 87 | 3 | 14 |
| Ras Gtpase | EgrG_000944700 | 36 | 3 | 5 |
| Hepatocyte Growth Factor Regulated Tyrosine | EgrG_000656400 | 81 | 3 | 2 |
| Hypothetical Protein | EgrG_000811100 | 19 | 3 | 42 |
| Hypothetical Protein | EgrG_000303200 | 29 | 3 | 1 |
| Alpha N Acetylgalactosaminidase | EgrG_000340500 | 48 | 3 | 2 |
| Hypothetical Protein | EgrG_000190300 | 76 | 3 | 1 |
| Nardilysin, Partial | EgrG_000921800 | 53 | 3 | 1 |
| Synaptotagmin Protein 4 | EgrG_000631500 | 77 | 3 | 1 |
| Nadp Dependent Malic Enzyme | EgrG_001145700 | 70 | 3 | 1 |
| Tegumental Antigen | EgrG_001001400 | 24 | 3 | 5 |
| Collagen Type I Ii Iii V Xi Alpha | EgrG_001190600 | 123 | 3 | 2 |
| Lactate Dehydrogenase A | EgrG_000660800 | 35 | 3 | 1 |
| Usp6 N Terminal Protein | EgrG_000165500 | 82 | 3 | 4 |
| L Lactate Dehydrogenase B Chain | EgrG_000634800 | 49 | 3 | 1 |
| Expressed Conserved Protein | EgrG_000950500 | 34 | 3 | 450 |
| Wd Repeat Containing Protein 86 | EgrG_000721400 | 43 | 3 | 34 |
| Hypothetical Protein | EgrG_000806200 | 27 | 3 | 1 |
| Phosphoglucomutase | EgrG_000943900 | 63 | 3 | 2 |
| Aquaporin-3 | EgrG_001190800 | 35 | 3 | 4 |
| Crumbs 1 | EgrG_000338500 | 48 | 3 | 1 |
| Casein Kinase I Gamma | EgrG_000345900 | 68 | 3 | 3 |
| Stam-Binding Protein-Like Protein | EgrG_000723000 | 43 | 3 | 2 |
| Rhophilin 2 | EgrG_000179600 | 88 | 3 | 1 |
| Ras-Related Protein Rab-5B | EgrG_000990600 | 22 | 3 | 7 |
| Serine:Threonine Protein Phosphatase 2A 65 Kda | EgrG_000598100 | 65 | 3 | 4 |
| Glycogen Debranching Enzyme | EgrG_000644500 | 176 | 3 | 1 |
| Hypothetical Protein | EgrG_000663300 | 34 | 3 | 5 |
| G1Y162 Protein | EgrG_000515900 | 17 | 3 | 4 |
| Thyroid Hormone Receptor Interactor 10 | EgrG_000125900 | 61 | 3 | 4 |
| Protein Tfg | EgrG_000635100 | 57 | 3 | 8 |
| Cyclin G Associated Kinase | EgrG_000077000 | 146 | 3 | 68 |
| Dynein Light Chain 1, Cytoplasmic | EgrG_000071700 | 12 | 3 | 6 |
| Expressed Protein | EgrG_001096600 | 44 | 3 | 5 |
| Cdc42 | EgrG_000809400 | 22 | 3 | 9 |
| ATP Binding Cassette Subfamily B Mdr:Tap | EgrG_000931700 | 128 | 3 | 3 |
| Tegumental Antigen | EgrG_001001800 | 19 | 3 | 1 |
| Glutathione Transferase | EgrG_000685900 | 24 | 3 | 11 |
| Monocarboxylate Transporter | EgrG_000119300 | 77 | 3 | 6 |
| Aldo Keto Reductase Family 1 Member B4 | EgrG_000155600 | 34 | 3 | 1 |
| Charged Multivesicular Body Protein | EgrG_000302000 | 23 | 3 | 34 |
| Protein Lin 7 B | EgrG_001073500 | 21 | 3 | 1 |
| Epididymal Secretory Protein E1 | EgrG_000682900 | 20 | 3 | 2 |
| Glycosyltransferase 14 Family Member | EgrG_000412000 | 50 | 3 | 1 |
| Gmp Synthase Glutamine Hydrolyzing | EgrG_001198000 | 81 | 3 | 2 |
| Tyrosine Protein Kinase Src64B | EgrG_000642300 | 41 | 3 | 4 |
| Leukotriene A 4 Hydrolase | EgrG_000972400 | 71 | 3 | 1 |
| Receptor Type Tyrosine Protein Phosphatase O | EgrG_001149600 | 35 | 3 | 118 |
| Actin Depolymerizing Factor | EgrG_000528900 | 16 | 3 | 1 |
| Diagnostic Antigen Gp50, Partial | EgrG_000564000 | 22 | 3 | 6 |
| Tbc1 Domain Family 4 | EgrG_000881900 | 68 | 3 | 1 |
| Beta Arrestin 1 | EgrG_000201100 | 64 | 3 | 22 |
| Anion Exchange Protein 3 | EgrG_000965200 | 105 | 3 | 16 |
| Cell Polarity Protein | EgrG_001110700 | 150 | 3 | 3 |
| Fibronectin Type Iii Domain-Containing Protein | EgrG_000948400.2 | 17 | 3 | 13 |
| 14-3-3 Protein Zeta | EgrG_001060100 | 29 | 3 | 2 |
| Sodium/Hydrogen Exchanger 2 | EgrG_001138300 | 85 | 3 | 4 |
| Amiloride Sensitive Amine Oxidase | EgrG_000530400 | 84 | 3 | 25 |
| T-Complex Protein 1 Subunit Alpha | EgrG_000826700 | 60 | 3 | 2 |
| Yl Amino Acid Transporter 2 | EgrG_000829200 | 59 | 3 | 2 |
| Glucose-6-Phosphate 1-Dehydrogenase | EgrG_000126400 | 60 | 3 | 4 |
| Aspartyl Aminopeptidase | EgrG_001083400 | 118 | 3 | 3 |
| Protein Disulfide Isomerase A6 | EgrG_001002700 | 54 | 3 | 3 |
| Aldo Keto Reductase Family 1 Member B4 | EgrG_000523800 | 36 | 3 | 5 |
| Tetraspanin | EgrG_001077400 | 24 | 3 | 19 |
| Expressed Protein | EgrG_001110600 | 113 | 3 | 1 |
| Calponin | EgrG_000595700 | 24 | 2 | 2 |
| Hypothetical Protein | EgrG_000952200 | 47 | 2 | 2 |
| Fras1 Related Extracellular Matrix Protein | EgrG_000176400 | 263 | 2 | 2 |
| Nascent Polypeptide Associated Complex Subunit | EgrG_001007300 | 30 | 2 | 3 |
| Exocyst Complex Component 3 | EgrG_000970300 | 85 | 2 | 1 |
| Metacestode Specific Membrane Protein 1 | EgrG_000353400 | 24 | 2 | 2 |
| Mitochondrial Sodium:Hydrogen Exchanger | EgrG_000546600 | 43 | 2 | 34 |
| Structural Maintenance Of Chromosomes Protein 2 | EgrG_000602100 | 149 | 2 | 5 |
| Ras Protein M Ras | EgrG_000952100 | 26 | 2 | 1 |
| Hypothetical Protein | EgrG_002052200 | 58 | 2 | 1 |
| Hypothetical Protein | EgrG_000411500 | 44 | 2 | 3 |
| Innexin Unc 9 | EgrG_000448500 | 57 | 2 | 6 |
| Tegumental Antigen | EgrG_001201000 | 27 | 2 | 1 |
| Subfamily S1A Unassigned Peptidase S01 Family | EgrG_001046200 | 36 | 2 | 6 |
| Fatty Acid-Binding Protein | EgrG_000551000 | 17 | 2 | 4 |
| Immunoglobulin | EgrG_000362000 | 41 | 2 | 20 |
| Adenylosuccinate Synthetase | EgrG_001056900 | 50 | 2 | 1 |
| Expressed Conserved Protein | EgrG_000372900 | 44 | 2 | 1 |
| Charged Multivesicular Body Protein 4A | EgrG_001133200 | 24 | 2 | 5 |
| Docking Protein 3 | EgrG_001014400 | 74 | 2 | 1 |
| Serine:Threonine Protein Phosphatase Pp1 Gamma | EgrG_000779500 | 43 | 2 | 3 |
| Niemann-Pick C1 Protein | EgrG_001107950 | 150 | 2 | 24 |
| Hypothetical Protein | EgrG_001058700 | 92 | 2 | 1 |
| Collagen Alpha 1V Chain | EgrG_000203400 | 171 | 2 | 15 |
| Armadillo Type Fold | EgrG_000222600 | 76 | 2 | 1 |
| Actin-Related Protein 2/3 Complex Subunit 1A | EgrG_000616500 | 35 | 2 | 2 |
| Rabb And C | EgrG_000173300 | 31 | 2 | 77 |
| Syntaxin Binding Protein 5 | EgrG_000642900 | 152 | 2 | 3 |
| Non-Lysosomal Glucosylceramidase | EgrG_000822700 | 112 | 2 | 5 |
| Lachesin | EgrG_000990100 | 44 | 2 | 1 |
| Dnaj Subfamily B Member | EgrG_000665400 | 28 | 2 | 19 |
| Cadmium Metallothionein Precursor | EgrG_000932700 | 20 | 2 | 30 |
| Cd63 Antigen | EgrG_000977700 | 26 | 2 | 5 |
| Guanine Nucleotide-Binding Protein Subunit Beta | EgrG_000612000 | 39 | 2 | 9 |
| Ferm Central Domain | EgrG_001079700 | 24 | 2 | 7 |
| Elongation Factor 2 | EgrG_000865300 | 94 | 2 | 2 |
| Protein Phosphatase 1 Regulatory Subunit 7 | EgrG_000429200 | 49 | 2 | 1 |
| Pfam-B_617 And Pfam-B_8201 Domain Containing Protein | EgrG_001086300 | 21 | 2 | 5 |
| Retinaldehyde Binding Protein | EgrG_001146700 | 40 | 2 | 1 |
| Expressed Conserved Protein | EgrG_000170100 | 30 | 2 | 82 |
| Innexin Unc 9 | EgrG_001000000 | 64 | 2 | 1 |
| Programmed Cell Death Protein | EgrG_000925800 | 15 | 2 | 2 |
| Expressed Conserved Protein | EgrG_000596300 | 25 | 2 | 1 |
| Proteasome Subunit Beta T Family | EgrG_001064900 | 23 | 2 | 3 |
| Tetraspanin | EgrG_000328400 | 20 | 2 | 5 |
| Camp Dependent Protein Kinase Catalytic Subunit | EgrG_000669700 | 46 | 2 | 2 |
| Predicted: Histone H4-Like | EgrG_000323100 | 11 | 2 | 1 |
| Spondin Extracellular Matrix Glycoprotein | EgrG_000929500 | 111 | 2 | 5 |
| Procollagen-Lysine,2-Oxoglutarate 5-Dioxygenase | EgrG_000443200 | 83 | 2 | 4 |
| Myosin Heavy Chain | EgrG_000920900 | 223 | 2 | 4 |
| Ras-Related Protein Rabd2A | EgrG_000255300 | 22 | 2 | 73 |
| Ras Protein Rab 8B | EgrG_001003300 | 28 | 2 | 2 |
| Guanine Nucleotide Binding Protein Gs Subunit | EgrG_000677700 | 44 | 2 | 4 |
| Resistance To Inhibitors Of Cholinesterase | EgrG_000226800 | 23 | 2 | 5 |
| Proteasome Prosome Macropain | EgrG_000590200 | 30 | 2 | 1 |
| Tetraspanin Similiar To Uroplakin 1 | EgrG_000989900 | 33 | 2 | 35 |
| Dynein Light Chain | EgrG_000941000 | 22 | 2 | 18 |
| Dynein Light Chain 2, Cytoplasmic | EgrG_000941100 | 11 | 2 | 9 |
| Reticulon 4 | EgrG_000685500 | 26 | 2 | 1 |
| Dual Specificity | EgrG_000352000 | 73 | 2 | 11 |
| Calcium Binding Protein 39 | EgrG_000203600 | 45 | 2 | 16 |
| Tetraspanin | EgrG_000354800 | 27 | 2 | 3 |
| Hypothetical Protein | EgrG_000233600 | 26 | 2 | 1 |
| Superoxide Dismutase [Cu-Zn] | EgrG_000638300 | 16 | 2 | 5 |
| Dynactin Subunit | EgrG_000871600 | 44 | 2 | 1 |
| Transmembrane 9 Superfamily | EgrG_000042800 | 68 | 2 | 14 |
| Uroporphyrinogen Decarboxylase | EgrG_000492300 | 44 | 2 | 4 |
| E3 Ubiquitin-Protein Ligase 8-Mar | EgrG_000154000 | 35 | 2 | 50 |
| Sn1-Specific Diacylglycerol Lipase Beta | EgrG_000642000 | 81 | 2 | 5 |
| 1 Acyl Sn Glycerol 3 Phosphate Acyltransferase | EgrG_000439000 | 50 | 2 | 6 |
| Lysyl Oxidase | EgrG_000217900 | 55 | 2 | 2 |
| Tetraspanin | EgrG_000355900 | 29 | 2 | 1 |
| Calcium Transporting Atpase | EgrG_001008200 | 117 | 2 | 4 |
| Mitogen Activated Protein Kinase | EgrG_000891000 | 43 | 2 | 1 |
| Protein Efr3 A | EgrG_000485700 | 93 | 2 | 2 |
| Fatty Acid-Binding Protein | EgrG_000417200 | 20 | 2 | 85 |
| Expressed Conserved Protein | EgrG_000042100 | 13 | 2 | 1 |
| C2 Calcium Lipid Binding Region Calb | EgrG_000320160 | 72 | 2 | 1 |
| Ndr | EgrG_001065500 | 40 | 2 | 6 |
| Dynein Light Chain | EgrG_000990800 | 11 | 2 | 1 |
| Ankyrin Repeat Domain Containing Protein | EgrG_000151300 | 76 | 2 | 10 |
| Dnaj Subfamily C B | EgrG_000993600 | 34 | 2 | 3 |
| Protein Of Unknown Function Duf284 Transmembrane Eukaryotic | EgrG_000800700 | 39 | 2 | 12 |
| Hypothetical Protein | EgrG_000757200 | 35 | 2 | 17 |
| Universal Stress Protein | EgrG_000779700 | 18 | 2 | 1 |
| Expressed Protein | EgrG_000172700 | 12 | 2 | 3 |
| Sorbin | EgrG_000411400 | 22 | 2 | 1 |
| Hypothetical Protein | EgrG_000174200 | 259 | 2 | 8 |
| T-Complex Protein 1 Subunit Theta | EgrG_000683800 | 59 | 2 | 3 |
| Exocyst Complex Component 5 | EgrG_000342100 | 84 | 2 | 2 |
| Antigenb | EgrG_000381200 | 10 | 2 | 14 |
| Leucine Rich Repeat Containing Protein 15 | EgrG_000500600 | 80 | 2 | 4 |
| Immunoglobulin Domain Containing Protein | EgrG_000318750 | 34 | 2 | 2 |
| Cysteine Rich Hydrophobic Domain 1 Protein | EgrG_000767900 | 22 | 2 | 3 |
| 6 Phosphofructokinase | EgrG_001128600 | 89 | 2 | 2 |
| Rhodopsin-Like Orphan Gpcr | EgrG_000633100 | 55 | 2 | 1 |
| Fatty Acid Amide Hydrolase 1 | EgrG_000743700 | 65 | 2 | 5 |
| Heat Shock 70 Kda Protein 4 | EgrG_000938600 | 61 | 2 | 2 |
| Sodium Dependent Neurotransmitter Transporter | EgrG_000107600 | 64 | 2 | 7 |
| Long Chain Fatty Acid Coenzyme A Ligase 5 | EgrG_000445600 | 80 | 2 | 7 |
| Actin 6A | EgrG_001191400 | 46 | 2 | 1 |
| Betaine Aldehyde Dehydrogenase | EgrG_000904200 | 74 | 2 | 1 |
| Zinc Transporter Zip8 | EgrG_000946600 | 60 | 2 | 7 |
| Dynein Light Chain 1, Cytoplasmic | EgrG_000071400 | 11 | 2 | 14 |
| Growth Factor Receptor Bound Protein 2 | EgrG_000587600 | 28 | 2 | 1 |
| Vesicle Associated Membrane Protein | EgrG_000528000 | 64 | 2 | 3 |
| Actin-Related Protein 2/3 Complex Subunit 2 | EgrG_000359700 | 37 | 2 | 15 |
| Alpha Tocopherol Transfer Protein | EgrG_000758100 | 37 | 2 | 13 |
| Multidrug Resistance Protein 1 | EgrG_000160300 | 28 | 2 | 10 |
| Beta Hexosaminidase Subunit Alpha | EgrG_000901900 | 59 | 2 | 1 |
| Expressed Protein | EgrG_000315600 | 23 | 2 | 1 |
| Ferric Chelate Reductase | EgrG_000487700 | 54 | 2 | 26 |
| Retinal Guanylyl Cyclase 2 | EgrG_000406000 | 83 | 2 | 1 |
| Neutral Amino Acid Transporter A | EgrG_001168200 | 33 | 2 | 5 |
| Sodium:Myo Inositol Cotransporter | EgrG_001173900 | 52 | 2 | 5 |
| Tetraspanin-6 | EgrG_000355500 | 29 | 2 | 17 |
| Hypothetical Protein | EgrG_000122100 | 13 | 2 | 2 |
| Zinc Finger Fyve Domain Containing Protein 19 | EgrG_000881500 | 31 | 2 | 1 |
| Beta D Xylosidase 2 | EgrG_000879900 | 92 | 2 | 1 |
| Pfam-B_1491 And Pfam-B_5139 Domain Containing Protein | EgrG_000773000 | 16 | 2 | 7 |
| Actin Interacting Protein 1 | EgrG_000994400 | 68 | 2 | 5 |
| T Complex Protein 1 Subunit Eta | EgrG_000576700 | 60 | 2 | 2 |
| Proto Oncogene Tyrosine Protein Kinase Receptor | EgrG_000469100 | 33 | 2 | 1 |
| Dnl2 Protein | EgrG_000946900 | 14 | 2 | 1 |
| Cyclin Dependent Kinase 1 | EgrG_001184700 | 65 | 2 | 2 |
| Heat Shock Protein 70B | EgrG_000733200 | 73 | 2 | 1 |
| Hypothetical Protein | EgrG_000358100 | 53 | 2 | 1 |
| Hypothetical Protein | EgrG_001085900 | 23 | 2 | 4 |
| Rhogap Domain Containing Protein | EgrG_000585200 | 74 | 2 | 24 |
| Folate Receptor Beta | EgrG_000751800 | 29 | 2 | 1 |
| Developmentally Regulated Gtp Binding Protein 1 | EgrG_000250600 | 36 | 2 | 2 |
| Expressed Conserved Protein | EgrG_001174100 | 23 | 2 | 3 |
| Neural Cell Expressed Developmentally | EgrG_000362800 | 118 | 2 | 5 |
| Collagen Type Xi Alpha 2 | EgrG_000524200 | 163 | 2 | 2 |
| Tubulin Beta 2C Chain | EgrG_000202600 | 58 | 2 | 11 |
| Dynamin 1 | EgrG_000878000 | 102 | 2 | 1 |
| Mitochondrial Import Receptor Subunit Tom34 | EgrG_000887500 | 44 | 2 | 1 |
| Hypothetical Protein | EgrG_000470500 | 82 | 2 | 4 |
| Calcium Activated Potassium Channel | EgrG_000135700 | 226 | 2 | 1 |
| T Complex Protein 1 Subunit Delta | EgrG_000909400 | 57 | 2 | 1 |
| Tau Tubulin Kinase 1 | EgrG_000922600 | 82 | 2 | 1 |
| Vacuolar Protein Sorting-Associated Protein | EgrG_000630700 | 13 | 2 | 1 |
| Lipase Maturation Factor | EgrG_000995800 | 70 | 2 | 1 |
| Importin-5 | EgrG_000063800 | 127 | 2 | 1 |
| Glycolipid Transfer Protein | EgrG_000827550 | 24 | 2 | 1 |
| Lipoxygenase Domain Containing Protein | EgrG_001180600 | 277 | 2 | 2 |
| Thioredoxin | EgrG_000666700 | 15 | 2 | 1 |
| Aminoacylase 1 | EgrG_000675100 | 49 | 2 | 1 |
| Stress Induced Phosphoprotein 1 | EgrG_000264900 | 64 | 2 | 10 |
| Rab14 Member Ras Oncogene Family | EgrG_000449500 | 28 | 2 | 1 |
| Excitatory Amino Acid Transporter | EgrG_000819900 | 46 | 2 | 1 |
| Rho Gtpase Activating Protein 1 | EgrG_000425300 | 55 | 2 | 25 |
| Ras-Related C3 Botulinum Toxin Substrate 2 | EgrG_000261400 | 21 | 2 | 3 |
| Charged Multivesicular Body Protein 5 | EgrG_001063800 | 24 | 2 | 10 |
