# Supplementary material for: Identification of Different Extracellular Vesicles in the Hydatid Fluid of Echinococcus granulosus and Immunomodulatory Effects of 110 K EVs on Sheep PBMCs
Source: Front Immunol. 2021 Feb 23;12:602717. doi: 10.3389/fimmu.2021.602717 (PMC7940240; doi:10.3389/fimmu.2021.602717)
Supplement: Supplementary file 4 [file Table_4.DOCX]

Table S4 miRNAs in different *E. granulosus* HF EVs

| **miR name** | **Sequence (5'-3')** | **Length** | **Read count (2 K EVs)** | **Read count (10 K EVs)** | **Read count (110 K EVs)** |
| --- | --- | --- | --- | --- | --- |
| egr-miR-71 | TGAAAGACGATGGTAGTGAGA | 21 | 623 | 3864 | 824738 |
| egr-let-7 | TGAGGTAGTGTTTCGAATGTCT | 22 | 32547 | 97203 | 174412 |
| egr-miR-9 | TCTTTGGTTATCTAGCTGTGTG | 22 | 82 | 763 | 117121 |
| egr-miR-7 | TGGAAGACTGGTGATATGTTGT | 22 | 3 | 0 | 54870 |
| egr-miR-2a | AATCACAGCCCTGCTTGGAACC | 22 | 9 | 1399 | 27291 |
| egr-miR-219 | TGATTGTCCATTCGCATTTCTTG | 23 | 75 | 643 | 26378 |
| egr-miR-190 | AGATATGTTTGGGTTACTTGGTG | 23 | 0 | 0 | 12395 |
| egr-miR-277 | TAAATGCATTTTCTGGCCCGTA | 22 | 0 | 0 | 12124 |
| egr-miR-4989 | AAAATGCACCAACTATCTGAGA | 22 | 0 | 0 | 10635 |
| egr-miR-125 | TCCCTGAGACCCTAGAGTTGTC | 22 | 0 | 0 | 9613 |
| egr-miR-10 | CACCCTGTAGACCCGAGTTTGA | 22 | 202 | 0 | 8216 |
| egr-miR-1 | TGGAATGTTGTGAAGTATGT | 20 | 0 | 0 | 5711 |
| egr-miR-8 | TAATACTGTTCGGTTAGGACGCC | 23 | 21 | 3 | 5356 |
| egr-miR-87-3p | GTGAGCAAAGTTTCAGGTGT | 20 | 5 | 0 | 4212 |
| egr-miR-124b-3p | TAAGGCACGCGGTGAATACC | 20 | 0 | 0 | 4178 |
| egr-miR-2c-3p | TCACAGCCAATATTGATGAA | 20 | 0 | 0 | 2895 |
| egr-miR-124b-5p | GTATTCTACGCGATGTCTTGGTA | 23 | 0 | 0 | 2691 |
| egr-miR-2b | TATCACAGCCCTGCTTGGGACA | 22 | 9 | 1 | 1538 |
| egr-miR-745 | TGCTGCCTGGTAAGAGCTGTGA | 22 | 0 | 0 | 838 |
| egr-miR-87-5p | CCACCTGTCATTTTGCTCGAACC | 23 | 0 | 0 | 427 |
| egr-miR-2c-5p | TCGTCAACATTGCCTGTAGACA | 22 | 0 | 0 | 232 |
| egr-miR-4991 | GATCCTGGAATCCAACCTCATT | 22 | 0 | 0 | 169 |
| egr-miR-124a | TAAGGCACGCGGTGAATGCCA | 21 | 25 | 21 | 78 |
| egr-miR-4988 | ACCTATCACACTTCAGTCC | 19 | 0 | 0 | 55 |
| egr-miR-153 | TTGCATAGTCTCATAAGTGCCA | 22 | 0 | 0 | 23 |
